# NLRP3 inflammasome of renal tubular epithelial cells induces kidney injury in acute hemolytic transfusion reactions

**DOI:** 10.1002/ctm2.373

**Published:** 2021-03-30

**Authors:** Zhixin Liu, Yaozhen Chen, Bing Niu, Dandan Yin, Fan Feng, Shunli Gu, Qunxing An, Jinmei Xu, Ning An, Jing Zhang, Jing Yi, Wen Yin, Xiangyang Qin, Xingbin Hu

**Affiliations:** ^1^ Department of Transfusion Medicine, Xijing Hospital Fourth Military Medical University Xi'an Shaanxi China; ^2^ School of Life Sciences Shanghai University Shanghai China; ^3^ Department of Hematology, Tangdu Hospital Fourth Military Medical University Xi'an Shaanxi China; ^4^ Division of Digestive Surgery, Xijing Hospital of Digestive Diseases Fourth Military Medical University Xian Shaanxi China; ^5^ Department of Chemistry, School of Pharmacy Fourth Military Medical University Xi'an Shaanxi China

**Keywords:** acute hemolytic transfusion reaction, heme, inhibitor, NLRP3 inflammasome, renal tubular epithelial cells

## Abstract

**Background:**

Blood transfusion, a common basic supporting therapy, can lead to acute hemolytic transfusion reaction (AHTR). AHTR poses a great risk to patients through kidney function damage in a short time. Previous reports found that heme from destroyed red blood cells impaired kidney function, and NLR family pyrin domain containing 3 (NLRP3) inflammasome was augmented in case of kidney injury. However, the detailed mechanism regarding whether NLRP3 inflammasome is involved in kidney function injury in AHTR is not fully understood yet.

**Methods:**

Hemolysis models were established by vein injection with human blood plasma or mouse heme from destroyed red blood cells. The injured renal tubular epithelial cells (RTECs) were evaluated by tubular damage markers staining in hemolysis models and in primary RTECs *in vitro*. The activation of NLRP3 inflammasome in RTECs by hemes was investigated by Western blot, ELISA, scanning electron microscopy, immunofluorescent staining, flow cytometry, and hemolysis models. NLRP3 gene knockout mice were employed to confirm these observations in vitro and in vivo. The binding between a novel inhibitor (66PR) and NLRP3 was affirmed by molecule docking and co‐immunoprecipitation. The rescue of 66PR on kidney function impairment was explored in murine hemolysis models.

**Results:**

We found that heme could activate NLRP3 inflammasome in RTECs to induce kidney function injury. NLRP3 gene knockout could prevent the damage of RTECs caused by hemes and recover kidney function in AHTR. Moreover, NLRP3 inflammasome chemical inhibitor, 66PR, could bind to NLRP3 protein and inhibit inflammasome activation in RTECs, which consequently relieved the injury of RTECs caused by hemes, and alleviated kidney function damage in the AHTR model.

**Conclusions:**

Hemes could activate NLRP3 inflammasome in RTECs, and a novel NLRP3 inflammasome inhibitor named 66PR relieved kidney function damage in AHTR. Our findings provided a new possible strategy to treat kidney function failure in AHTR.

## Graphical abstract

The released hemes in acute hemolytic transfusion reaction activates NLRP3 inflammasome through potassium efflux and ROS signal in renal tubular epithelial cells.

Hemes from lysed RBCs induces pyroptosis and IL‐1β release of renal tubular epithelial cells in acute hemolytic transfusion reaction.

The 66PR chemical inhibits hemes’ activation of NLRP3 inflammasome in renal tubular epithelial cells to protect kidney function.

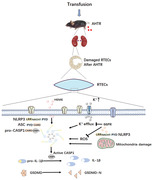

**Abbreviations**
AHTR: acute hemolytic transfusion reaction
AQP1: aquaporin‐1
ASC: apoptosis associated speck‐like protein containing a caspase recruitment domain
CK18: cytokeratin‐18
CMXRos: chloromethyl‐X‐rosamine
Co‐IP: co‐immunoprecipitation
GSDMDC1: gasdermin domain containing 1
HTR: hemolytic transfusion reaction
NGAL: neutrophil gelatinase associated lipocalin
NLRP3: nucleotide binding oligomerization domain‐like receptor family pyrin domain containing 3
RBCs: red blood cells
ROS: reactive oxygen species
RTECs: renal tubular epithelial cells
SGLT2: sodium‐glucose co‐transporter 2
Tim‐1: T cell immunoglobulin and mucin 1

## 1 BACKGROUND

Blood transfusion is a common therapy in clinical settings. However, the incompatible red blood cells (RBCs) and, more rarely, a large volume of incompatible plasma are common causes of hemolytic transfusion reactions (HTRs).^1^, ^2^ Delayed HTRs are caused by a secondary immune response to the donor's RBCs. Acute HTRs (AHTRs) occur within 24 h after administration of a blood product.^3^, ^4^ Once an AHTR occurs, a mass of heme is produced. When heme cannot be evacuated by haptoglobin and heme oxygenase, excessive heme leads to damage of organs,^5^ such as the heart, lung, brain, gut, pancreas, liver, and kidney^6^; therefore, an AHTR can result in death.

Under normal conditions, when blood circulates in the glomerulus, crude urine is collected and passed through the renal tubules in the kidney. Renal tubular epithelial cells (RTECs) can then reabsorb water, glucose, amino acids, urea, and electrolytes. Subsequently, terminal urine is produced and excreted. If the RTECs are impaired, effective reabsorption cannot occur, leading to kidney failure, which in turn results in abnormal physiological life activity.^7^ When high concentrations of heme circulate through the kidney in an AHTR, various types of cells, such as macrophages, vascular endothelial cells, mesenchymal cells, podocytes, and tubular epithelial cells, are constantly in contact with heme, leading to heme‐induced toxicity.^8^ Ferriheme causes acute kidney failure, and heme oxygenase inhibition promotes apoptosis of RTECs.^5^, ^9^ However, the mechanism by which heme induces RTEC damage is not fully understood.

Studies have reported the abnormal expression of major components of NLRP3 inflammasome in renal tissue change in nephritis or renal injury.^10^, ^11^, ^12^ NLRP3 is an essential inflammasome, and can be activated by virous stimuli.^13^, ^14^, ^15^ The NLRP3 inflammasome contains NLRP3, an apoptosis‐associated speck‐like protein containing a caspase recruitment domain (ASC), and pro‐caspase‐1. The NLRP3 inflammasome cleaves pro‐caspase‐1 into p20 and p10, inducing the maturation and release of IL‐1β and IL‐18.^16^ The NLRP3 inflammasome activation also leads to pyroptosis.^17^ Some studies have reported that heme activates the NLRP3 inflammasome in macrophages.^18^, ^19^ Other studies have found that RTECs express a functional NLRP3 inflammasome in renal diseases.^20^, ^21^, ^22^ However, there is insufficient detailed evidence to determine whether heme activates NLRP3 inflammasomes in RTECs.

If we demonstrate that the NLRP3 inflammasome is deeply involved in heme‐induced RTEC impairment, then the inhibition of NLRP3 inflammasome will undoubtedly be important in the control of kidney function failure in hemolysis. NLRP3 inhibitors have been shown to be effective in treating diabetes, cancer, and infections.^23^ Coll *et al* found that a compound named MCC950 directly binds to the Walker B motif of NLRP3 NACHT domain, thereby blocking adenosine triphosphate hydrolysis and inhibiting NLRP3 inflammasome recombination and activation.^24^, ^25^ Another chemical compound, CY‐09, was also shown to inhibit the activation of NLRP3 inflammation.^26^ We previously reported that a small molecule named 66PR was a novel NLRP3 inhibitor that could inhibit the progression of inflammatory bowel diseases,^27^ which prompted us to further investigate the possibility and potential of applicating 66PR to restore the function of RTEC in AHTRs.

The current study focused on heme, especially heme from hemolysis, to elucidate how NLRP3 inflammasomes were activated in RTECs. Based on the activation mechanism, we also tried to inhibit the NLRP3 pathway to maintain kidney function in an animal model of hemolysis.

## 2 MATERIALS AND METHODS

### 2.1 Mice and animal model

Adult wild‐type C57BL/6 mice (8‐12 weeks old) were purchased from the Animal Resources Center of the Fourth Military Medical University (Xi'an, China). NLRP3 gene knockout mice (NLRP3^−/−^, C57BL/6 stains) were what had been described,^28^, ^29^ and littermates were used as controls. All mice were maintained under a special pathogen‐free environment and randomly assigned in experiments. The mice experimental protocols were approved by the Animal Care Committee of the Fourth Military Medical University of China.

Two types of hemolysis models were used in this study. One model was induced by intravenous injection with human blood plasma (5 μL/g; informed consent was obtained from volunteer donors). To ensure hemolysis, C57BL/6 murine red blood cells were mixed with human plasma to detect agglutination. Murine peripheral blood (20 μL) was collected and washed three times with saline. Murine red blood cells (5 μL) were added to 100 μL of human plasma and centrifuged. After centrifugation, the samples were gently shaken and observed. Further experiments were performed only when agglutination was confirmed in vitro. The agglutination in vitro is shown in Figure S1A,B. Another model was developed by intravenous injection with murine heme (10 mL/kg; murine RBCs were freeze–thawed and centrifuged to obtain heme). In both models, hemoglobinuria was observed in mice at 1–4 h post‐injection. After 4 h, peripheral blood and urine samples were collected. RBC counts in the animals’ peripheral blood were determined using an automatic hematology analyzer (SYSMEX XP‐100, Kobe, Japan). Free hemoglobin in the peripheral blood and urine was analyzed using Trinder's reaction (plasma‐free hemoglobin assay kit; Beijing Ruilda Biotechnology Co. Ltd., China). The creatinine (CREA), blood urea nitrogen (BUN), and lactate dehydrogenase (LDH) levels in the blood were measured using an automatic biochemical analyzer (HITACHI7600, Tokyo, Japan). Coomb's test was performed using a commercial kit (Anti‐IgG&C3d, Shanghai Pharmacy Blood, Co., Ltd., Shanghai, China).

### 2.2 Reagents

Anti‐bodies used for murine cell immunoblot analysis were: IL‐1β (#12426, Cell Signaling Technologies, the dilution ratio [the following are the same] was 1:1000), NLRP3 (#AG‐20B‐0014, Adipogen, 1:1000), Caspase‐1p20 (#AG‐20B‐0042, Adipogen, 1:1000), alpha Tubulin (#66031‐1‐1 g, Proteintech, 1:1000), ASC (#AG‐25B‐0006, Adipogen, 1:1000), GSDMDC1 (#sc‐393656, Santa Cruz Biotechnology, 1:100), Anti‐biotin (#ab53494, Abcam, 1:100) RP Goat Anti‐Mouse IgG Antibody (#EK010, Zhuangzhibio, 1:5000), and HRP Goat Anti‐Rabbit IgG Antibody (#EK020, Zhuangzhibio, 1:5000). Anti‐mouse antibodies used for immunofluorescent staining analysis were: Cytokeratin‐18 (CK18) (#10830‐1‐AP, Proteintech,1:200), Aquaporin‐1 (AQP1, #20333‐1‐AP, Proteintech, 1:200), Villin (#16488‐1‐AP, Proteintech, 1:200), Sodium‐glucose co‐transporter 2 (SGLT2) (#ab37296, Abcam, 1:200), Tim‐1 (#ab47653, Abcam, 1:200), NGAL (#ab63929, Abcam, 1:200), GSDMDC1 (#sc‐393656, Santa Cruz Biotechnology, 1:50), Cy3 Goat Anti‐MouseIgG Antibody (#EK012, Zhuangzhibio, 1:400), Alexa Fluor 488 Goat Anti‐ Mouse IgG Antibody (#EK011, Zhuangzhibio, 1:400), Cy3 Goat Anti‐Rabbit IgG Antibody (#EK022, Zhuangzhibio, 1:400), Alexa Fluor 488 Goat Anti‐ Mouse IgG Antibody (#EK021, Zhuangzhibio, 1:400).

Anti‐human antibodies used for immunoblot analysis were:IL‐1β (#ab9722, Abcam, 1:1000), NLRP3 (#AG‐20B‐0014, Adipogen,1:1000), Caspase‐1p20 (#AG‐20B‐0048, Adipogen, 1:1000), alpha Tubulin (#ab7291, Abcam, 1:1000), ASC (#AG‐25B‐0006, Adipogen, 1:1000), GSDMDC1 (#ab228824, Abcam, 1:1000), HRP Goat Anti‐Mouse IgG Antibody (#EK010, Zhuangzhibio, 1:4000), HRP Goat Anti‐Rabbit IgG Antibody(#EK020, Zhuangzhibio, 1:4000). Anti‐human antibodies used for immunofluorescent staining analysis were: Tim‐1 (the same as anti‐mouse, 1:200), NGAL (the same as anti‐mouse, 1:200), GSDMDC1 (#ab228824, Abcam, 1:200), Cy3 Goat Anti‐Mouse IgG Antibody (#EK012, Zhuangzhibio, 1:400), Cy3 Goat Anti‐Rabbit IgG Antibody (#EK022, Zhuangzhibio, 1:400).

### 2.3 HK‐2 cells, primary RTECs prepare and chemical heme treatment

The HK‐2 cells were obtained from the laboratory of Kidney Department, Xiijng Hospital and maintained in DMEM/F12 medium (Gibco, USA) with 5% CO_2_ at 37°C. A part of the extracted renal tissue from experimental C57BL/6 mice was used to isolate primary RTECs. Briefly, the cortex area of the kidney was cut into pieces of 1 mm,^3^ and the cells were isolated by incubation with 1 mg/mL collagenase type‐I (C1‐28, Sigma‐Aldrich) for 45 min at 37°C. The RBCs were removed by lysis. The cell suspension was collected after filtration through a 60‐mesh screen. RTECs were separated from the cell suspension using 180‐mesh screens. RTECs were maintained in DMEM/F12 medium containing 10% FBS and 5% CO_2_ at 37°C. Primary RTECs were determined by immunostaining with anti‐CK18, anti‐AQP1, anti‐Villin, and anti‐SGLT2 (Figure S1C,D). Chemical heme was freshly prepared with NaOH and hemin (#51280, Sigma‐Aldrich) in 1 mL culture solution (5 mmol/L).

### 2.4 IL‐1β level evaluation

Experiments to analyze IL‐1β secretion were performed with 2 × 10^5^ cells, and the cellular supernatants were collected for ELISA analysis (Mouse IL‐1β, #P16807, R & D; Human IL‐1β, #P250505, R & D, USA). All measurements were performed according to the manufacturer's instructions.

### 2.5 Western blot analysis

Cells were lysed in a radio immunoprecipitation assay buffer (#P0013C, Beyotime, China). Protein concentrations were quantified using the BCA Protein Assay Kit (#23225; Pierce, Rockford, IL, USA). Proteins in the supernatant of the cell culture were concentrated by 10% (v/v) trichloroacetic acid (TCA). Then, the precipitates were collected and washed three times with ethanol‐acetone (1:1, v/v). The precipitates were resuspended in a loading buffer.

Equal amounts of protein from different groups were loaded onto SDS‐PAGE and transferred onto PVDF membranes. The membranes were incubated with antibodies against NLRP3, caspase 1, GSDMD, ASC, IL‐1β, and α‐tubulin (as described above) for overnight, and then incubated with appropriate secondary HRP‐conjugated antibodies. Blots were detected using a chemiluminescence detection kit (#34077, Thermo Scientific, USA). Western blots were performed five times for each experiment. We used anti‐mouse a‐tubulin or anti‐human a‐tubulin unless otherwise specified, as the incubation with antibody was performed for each murine and human protein. Band intensities were determined based on alpha‐tubulin unless otherwise specified using Image J software (1.42q, Wayne Rasband, National Institutes of Health, USA).

### 2.6 Morphological analysis

#### 2.6.1 Hematoxylin‐eosin staining of kidney sections and analysis

After mice were killed, the kidneys were collected to prepare 8‐μm paraffin sections, and were stained with the standard Hematoxylin‐eosin (H&E) protocol. Semiquantitative histologic scoring was performed by two individuals independently with a blinded manner, according to a previous study.^30^ They randomly selected five slides of kidney sections from five mice and observed five microscopic fields per slide. The slides were observed at 200× magnification. The tubular cell necrosis scores were obtained using a severity index that assigns points (0 to 3) for pathological changes of renal tubules, according to the following criteria: for the extent of interstitial infiltrates, interstitial edema, tubular dilation, and atrophy were looked as absent (0), involving less than 25% of the section area with mild focal separation of tubules (1), involving 26–50% of the section area, with diffuse mild separation of tubules, and with flattening of the tubular epithelium (2), and involving more than 50% of the section area, containing tubular basement membranes diffusely separated by the thickness of a tubular epithelial cell, and the diameters of tubules exceeded those of glomeruli (3).

#### 2.6.2 Scanning electron microscopy

Cells were seeded at 5 × 10^4^ cells per well with glass slides and rested overnight for proper attachment. The cells were then treated with heme. After 4 h, the cell supernatants were removed, and the samples were fixed with 3% glutaraldehyde for 24 h. The samples were cleaned twice with distilled water, then dehydrated with an acetonitrile gradient in sequence (50% acetonitrile 10–15 min, 70% acetonitrile 10–15 min, 90% acetonitrile 10–15 min, 100% acetonitrile 10–15 min three times). The samples were then dried in a vacuum. Then, the samples were pasted onto the sample tables to spray gold. Finally, the samples were imaged with a voltage of 5 kV using a scanning electron microscope (Olympus N300M, Shinjuku‐ku, Tokyo, Japan). Once the images were taken, the pyroptotic pores were counted by two individuals independently in a blinded manner. They randomly selected five pictures and recorded the number of pores.

#### 2.6.3 Immunofluorescence staining and laser confocal microscopy

Kidney sections from animals were prepared according to the standard protocol (Leica CM1860, Wetzlar, Germany). Briefly, after mice sacrificed, the kidneys were collected, embedded in OCT, and stored at ‐20°C. Cryosections of the kidney were made using an 8 μm freezing microtome before staining. Kidney sections or cultured cells in dishes (# 801002, NEST) were washed twice with sterile PBS, fixed with 4% paraformaldehyde (PFA), and blocked in 5% BSA. The cells or kidney sections were then incubated overnight with primary antibodies, including anti‐GSDMDC1, anti‐Tim‐1, anti‐NGAL, anti‐CK18, anti‐AQP1, anti‐villin, or anti‐SGLT2. Secondary fluorescent antibodies were added for 1 h, and DAPI (#D9542, Sigma–Aldrich) was used for nuclear counterstaining. The samples were imaged using a laser confocal system (Nikon C2, Tokyo, Japan). Quantitative analysis was performed using Image J software (1.42q, Wayne Rasband, National Institutes of Health, USA).

### 2.7 Potassium level, mitochondria damage, ROS, and viability assay

Potassium level: HK‐2 cells were cultured in 6‐well plates, treated with heme, and inhibited for 4 h. Then, the supernatant was collected to measure potassium levels using a kit (Abacam, #ab252904, UK). Mitochondria damage: Briefly, the HK‐2 cells in different groups were incubated at 37°C for 15 min with 200 nmol/L Mito‐Tracker Red CMXRos (#C1049, Beyotime, China) and were collected and detected by flow cytometry (BD, FACS Canto II plus, United States). Intracellular ROS levels: According to the Reactive Oxygen Species Assay Kit (#S0033S, Beyotime, China), the HK‐2 cells in different groups were incubated at 37°C for 15 min with 10 μmol/L DCFH‐DA (included in #S0033S, Beyotime, China), and flow cytometry was used to detect intracellular ROS levels. For the viability assay, HK‐2 cells in different groups were cultured in 96‐well plates, treated with heme, and inhibited for 4 h. Then, cell viability was assessed using the kit (Boster Biological Technology Co. Ltd., Wuhan, China). N‐acetyl‐l‐cysteine (NAC) (#A9165, Sigma‐Aldrich), an inhibitor of ROS, was used to detect the changes in ROS and cell viability after different stimulations.

### 2.8 Synthesis and biotinylation of 66PR compound and molecule docking

The synthesis of 66PR compound and the characterization data were previously described.^27^ 66PR compound was linked with biotin. Molecular docking was performed between the PDBID:6NPY of NLRP3 and 66PR using Discovery Studio 2019 software.

### 2.9 Co‐immunoprecipitation

The cells were treated with biotin‐66PR. After treatment, the cells were collected to prepare proteins and co‐immunoprecipitation was performed using the Pierce co‐immunoprecipitation (Co‐IP) kit (Thermo Scientific, USA) according to the manufacturer's instructions. In brief, 20 μg of anti‐biotin was added directly to the resin in the spin column. The column was capped and incubated at room temperature for 90–120 min using a rotating body or mixer. After the antibody was immobilized, the protein extracts (500μg) were added to the resin, followed by overnight incubation at 4°C. Following the elution of Co‐IP samples, SDS‐PAGE samples were prepared and further immunoblotted with anti‐NLRP3 antibody. The control was IgG isotype (#ab109489, Abcam, 1:1000).

### 2.10 Treatment of hemolysis‐affected mice with chemical inhibitor

66PR (5 mg/kg) and MCC950 (5 mg/kg, #5.38120, Sigma–Aldrich) were administered intraperitoneally to hemolysis‐affected mice. The control hemolyzed animals received PBS. After 4 h, peripheral blood was collected for cell counts and biochemical tests. After sacrifice, partial kidney tissues were collected from mice for paraffin sections and frozen sections.

### 2.11 Statistical analysis

All in vitro experiments were repeated at least three times, and in vivo experiments included the indicated number of mice. Values are presented as the mean ± SD. Two‐group comparisons were performed using Student's t‐test. *P*‐values less than .05 were considered significant. Statistical analyses were performed using SPSS version 16.0 (Chicago, IL, USA).

## 3 RESULTS

### 3.1 RTECs were directly impaired by heme

**FIGURE 1:**
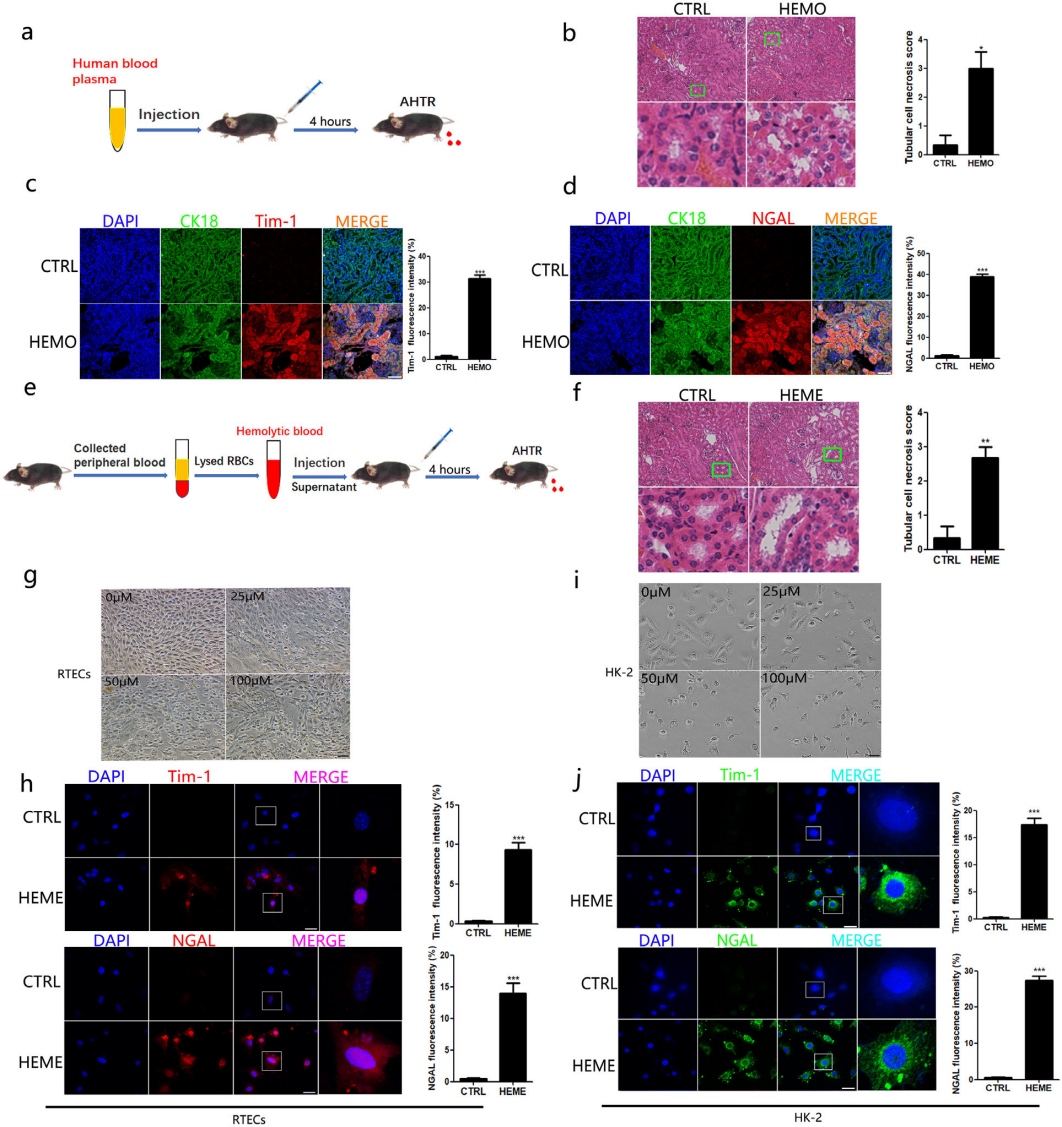
**Hemolysis caused renal tubular epithelial cells injury**. A, The scheme of mice hemolysis model with transfusion. Mice were transfused with human plasma (5 μL/g) via caudal vein, as the group of hemolysis. The control group was injected with equal amount of PBS. After 4 h, mice were sacrificed and kidneys were collected. B, The H&E staining of kidney section from hemolysis and control mice (bar = 200μm), and the tubular cell necrosis scores. The lower panel was enlarged from the green box of upper panel to show the tubular damage region. HEMO: the group of hemolysis; CTRL: the control group. C and D, The detection of TIM‐1 and NGAL by laser confocal microscope and quantity of RTECs containing TIM‐1^+^ foci. bar = 200μm. HEMO: the group of hemolysis; CTRL: the control group. E, The scheme of mice hemolysis model with heme from destroyed RBCs. Mice were injected with supernatant of lysated mice blood (10 μL/g) via caudal vein, as the group of hemolysis. The control group was injected with equal amount of PBS. After 4 h, mice were sacrificed and kidneys were collected. F, The H&E staining of kidney section from hemolysis and control mice (bar = 100 μm), and the tubular cell necrosis scores. The lower panel was enlarged from the green box of upper panel to show the tubular damage region. HEME: the group of hemolysis; CTRL: the control group. G and I, Primary RTECs and HK‐2 cells were observed under light microscope, after stimulated for 4 h with different concentration of chemical heme. (bar = 50μm). H and J, Primary RTECs and HK‐2 cells were stimulated for 4 h with 50 μM chemical heme. The detection of TIM‐1 and NGAL by laser confocal microscope (bar = 50μm) and quantity of containing TIM‐1^+^ foci and NGAL^+^ foci. The rightmost panel was enlarged from the white box of neighbor panel. DAPI: blue), CK18: green, TIM‐1: red, NGAL: red. Each data represented five mice per group. The quantity counts are shown as mean±SD. ^*^ *P* < .05; ^**^ *P* < .01; and ^***^ *P* < .001

To investigate whether RTECs were directly impaired by heme, we first established a murine model of AHTR through plasma transfusion (Figure 1A). After transfusion, the RBC count became lower (Figure S2A) and free hemoglobin level increased in the peripheral blood (Figure S2B) demonstrated that hemolysis occurred in mice. Furthermore, there were more poikilocytes in the blood smears from the hemolysis mice than those from the control (Figure S2C,D). Direct Coomb's test also indicated that the RBCs from hemolysis‐affected mice showed a positive reaction, while those from control mice showed a negative reaction (Figure S2E). Moreover, there was more free hemoglobin in the urine from the hemolysis group than the control group (Figure S2F,G). These data demonstrated that these mice underwent acute hemolysis after transfusion. Additionally, CREA (Figure S2H), BUN (Figure S2I), and LDH (Figure S2J) were significantly increased in the hemolysis group than those in the control group. These data suggest that AHTRs cause serious damage to kidney function. Pathological analysis of kidney tissue sections revealed that some RTEC regions were necrotic in the hemolysis group, and the RTEC region necrosis scores were higher in the hemolysis group than in the control group (Figure S1B). Because RTECs are instrumental in kidney reabsorption, we evaluated whether these cells were injured when hemolysis occurred. As shown in Figure 1C,D, more fluorescence signals due to T‐cell immunoglobulin and mucin domain 1 (TIM‐1) and neutrophil gelatinase‐associated lipocalin (NGAL), markers of renal tubular damage, were observed in the kidney tissue sections of the hemolysis group than in those of the control group. The results of western blotting also showed that the levels of TIM‐1 and NGAL were significantly increased in the hemolysis group than those in the control group (Figure S2N). These results implied that an AHTR resulted in RTEC injury.

Because hemolysis could produce large amounts of heme, we directly used the supernatant from destroyed murine RBCs to challenge the mice, as shown in Figure 1E. We found that heme from RBCs induced higher levels of CREA (Figure S2K), blood urea nitrogen (Figure S2L), and LDH (Figure S2M), which indicated that heme triggered kidney function injury. Furthermore, once hemes from RBCs were administered in vivo, similar damage morphology was observed in the kidney tissue sections (Figure 1F). Altogether, these observations demonstrate that RTECs are injured by hemes from RBCs in vivo.

Next, we investigated whether hemes directly injured the RTECs. After adding chemical hemes to the RTECs from the primary culture, more injured cells (above 50 μM hemes) were observed under a light microscope (Figure 1G), and immunofluorescence staining demonstrated that the damage markers exhibited stronger signals (Figure 1H). Moreover, when HK‐2 cells were co‐cultured with chemical hemes, similar morphological phenomena were observed (Figure 1I,J). Taken together, these data demonstrate that hemes can directly impair RTECs.

### 3.2 Hemes activated NLRP3 inflammasome to damage RTECs

**FIGURE 2:**
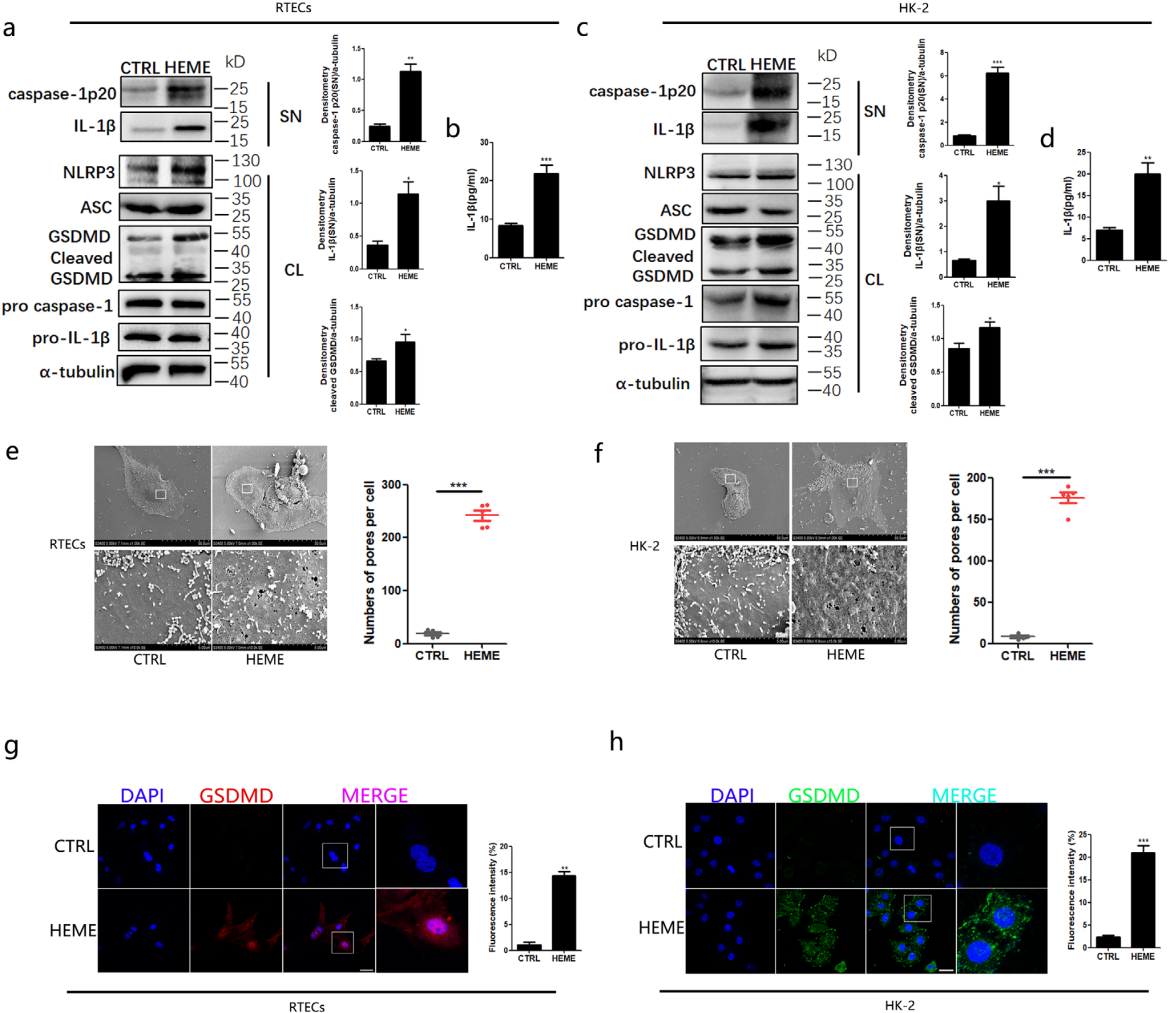
**Heme injured renal tubular epithelial cells through NLRP3 inflammasome activation in vitro**. A, Primary RTECs were stimulated for 4 h with 50μM chemical hemes. Western blot analysis for NLRP3, pro‐caspase1, caspase1p20, pro‐IL‐1β, IL‐1β, and GSDMD in RTECs after challenge. Total RTECs lysates (CL) and supernatants (SN) using indicated antibodies were detected after stimulation. B, ELISA analysis of IL‐1β in primary RTECs supernatants. C, HK‐2 cells were stimulated for 4 h with 50 μM chemical hemes. Western blot analysis for NLRP3, pro‐caspase1, caspase1p20, pro‐IL‐1β, IL‐1β, and GSDMD in HK‐2 cells after challenge. Total HK‐2 lysates (CL) and supernatants (SN) using indicated antibodies were detected after stimulation. D, ELISA analysis of IL‐1β in HK‐2 cells supernatants. E and F, Scanning electron microscopy observation of primary RTECs and HK‐2 cells (Magnification: the upper = 1000×; the lower = 10 000×) pyroptotic pore numbers after 50 μM chemical hemes treatment. The lower panel was enlarged from the white marked area of upper panel. G and H, The observation of nuclei (DAPI, blue) and GSDMD (red) foci of RTECs and HK‐2 cells by laser confocal microscope (bar = 50μm) and quantity of primary RTECs containing GSDMD^+^ foci. The rightmost panel was enlarged from the white box of neighbor panel. Data shown represent the mean ± SD from at least triplicate measurements. ^*^ *P* < .05; ^**^ *P* < .01; ^***^ *P* < .001

Bulks of evidence have reported that inflammasomes play a key role in kidney damage. To determine how hemes damage primary RTECs, we turned to the NLRP3 inflammasome. We first identified whether the NLRP3 inflammasome was present in primary RTECs. The NLRP3 inflammasome components, including NLRP3, ASC, and pro‐caspase‐1, were detected in RTECs from mice after a chemical heme treatment (Figure 2A). According to the caspase‐1p20 level in the supernatant of primary RTECs stimulated by hemes, the pro‐caspase‐1 protein was successfully cleaved (Figure 2A). We also observed that IL‐1β levels increased in the supernatant of primary RTECs stimulated by chemical hemes (Figure 2B). To further confirm this finding, we employed HK‐2 cells to conduct the investigation and obtained similar results (Figure 2C,D). Further, we found that chemical hemes resulted in the formation of pores on the surface of primary RTECs and HK‐2 cells under electron microscopy (Figure 2E,F). Here, the GSDMD‐induced pores demonstrated in our study were approximately 50 nm in diameter, which is much smaller than the pores on MCF‐7 cells (approximately 700 nm in diameter, measured through atomic force microscopy).^31^ However, they are larger than those of tumor cells (30 nm under a scanning electron microscope)^32^ and bone marrow‐derived macrophages (∼18 nm).^33^ Immunofluorescence analysis revealed that the percentage of GSDMD‐N (the executive molecule in pyroptosis) foci significantly increased in primary RTECs and HK‐2 cells after chemical heme stimulation (Figure 2G,H). Meanwhile, western blotting showed that cleaved GSDMD was obviously increased under heme stimulation in primary RTECs and HK‐2 cells (Figure 2A,C). These data demonstrated that hemes activated the NLRP3 inflammasome in RTECs in vitro.

**FIGURE 3:**
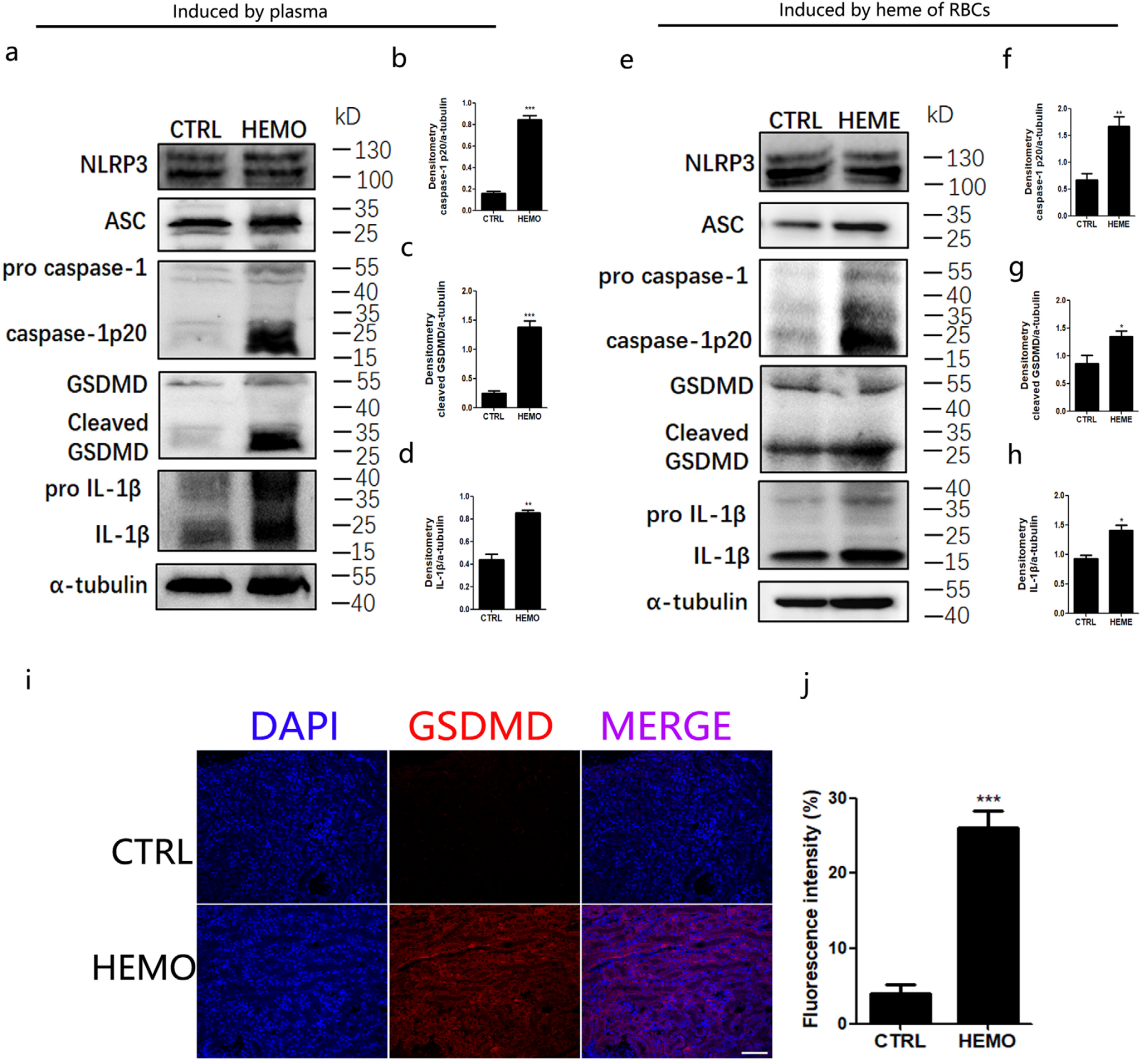
**NLRP3 inflammasome was activated by hemes in the murine models of hemolysis**. The animal model of hemolysis was established by transfused with human plasma as mentioned above, then followed by (A–D, I–J). A, Primary RTECs were isolated from transfusion mice and cultured for 2 h at 37°C. Proteins were prepared from the mixture of cells and the supernatant to perform the western blot of NLRP3, ASC, Caspase1, IL‐1β, and GSDMD. B–D, Densitometry analyses of caspase‐1, GSDMD, and IL‐1β based on (A). The animal model of hemolysis was established by injection of mice heme from destroyed RBCs, followed by (E–H). E. Western blot analysis for indicated proteins in primary RTECs from mice. F‐H, Densitometry analyses of IL‐1β, Caspase‐1, and GSDMD based on (E). I, The observation of GSDMD (red) foci of RTECs from the animal models of hemolysis transfused with human plasma by laser confocal microscope. J, Quantity of RTECs containing GSDMD^+^ foci, according to (I). Each data represented five mice per group, and quantity data are shown as mean ± SD. ^*^ *P* < .05; ^**^ *P* < .01; ^***^ *P* < .001

Next, we explored whether the NLRP3 inflammasome was activated in vivo after hemolysis. As shown in Figure 3A–D, more caspase‐1p20, IL‐1β, and GSDMD proteins were found in the RTECs of mice transfused with human blood plasma than in those of the controls. As expected, similar observations were noted in the RTECs of mice injected with hemes from murine destroyed RBCs (Figure 3E‐H). We also found more immunofluorescence signals of GSDMD‐N in RTECs from hemolysis‐affected animal model kidneys than in those from the controls (Figure 3I, J), suggesting that more pyroptosis of RTECs was induced by hemolysis. Altogether, these results suggested that hemes activated the NLRP3 inflammasome in RTECs in vivo.

The NLRP3 inflammasome is classically activated by the outflow of potassium (K^+^), mitochondrial damage, and reactive oxygen species (ROS).^16^ Here, we also investigated the mechanism of NLRP3 inflammasome activation in HK‐2 cells following heme stimulation. We found that the K+ concentration in the supernatant of HK‐2 cells increased after chemical heme challenge (Figure S3A). Moreover, when potassium was added to the culture system of HK‐2 cells stimulated by chemical hemes, IL‐1β, and cleaved caspase‐1 levels were significantly decreased (Figure S3B–D). These data indicated that the outflow of K^+^ was involved in the activation of the NLRP3 inflammasome in HK‐2 cells via heme. When we analyzed the mitochondrial membrane potential and ROS by fluorescent probes after chemical heme administration, we found that the mitochondrial membrane potential decreased (Figure S3E), while the ROS levels were upregulated dramatically (Figure S3F) in HK‐2 cells. We also observed that the ROS inhibitor, NAC, decreased the ROS level (Figure S3G), and the cell viability increased (Figure S3H) in chemical heme treated HK‐2 cells. Altogether, these data suggest that the K^+^ concentration, mitochondrial injury, and presence of ROS are involved in the activation of the NLRP3 inflammasome in renal tubular epithelial cells resulting from heme.

### 3.3 NLRP3 gene knockout could prevent the damage of heme in RTECs

**FIGURE 4:**
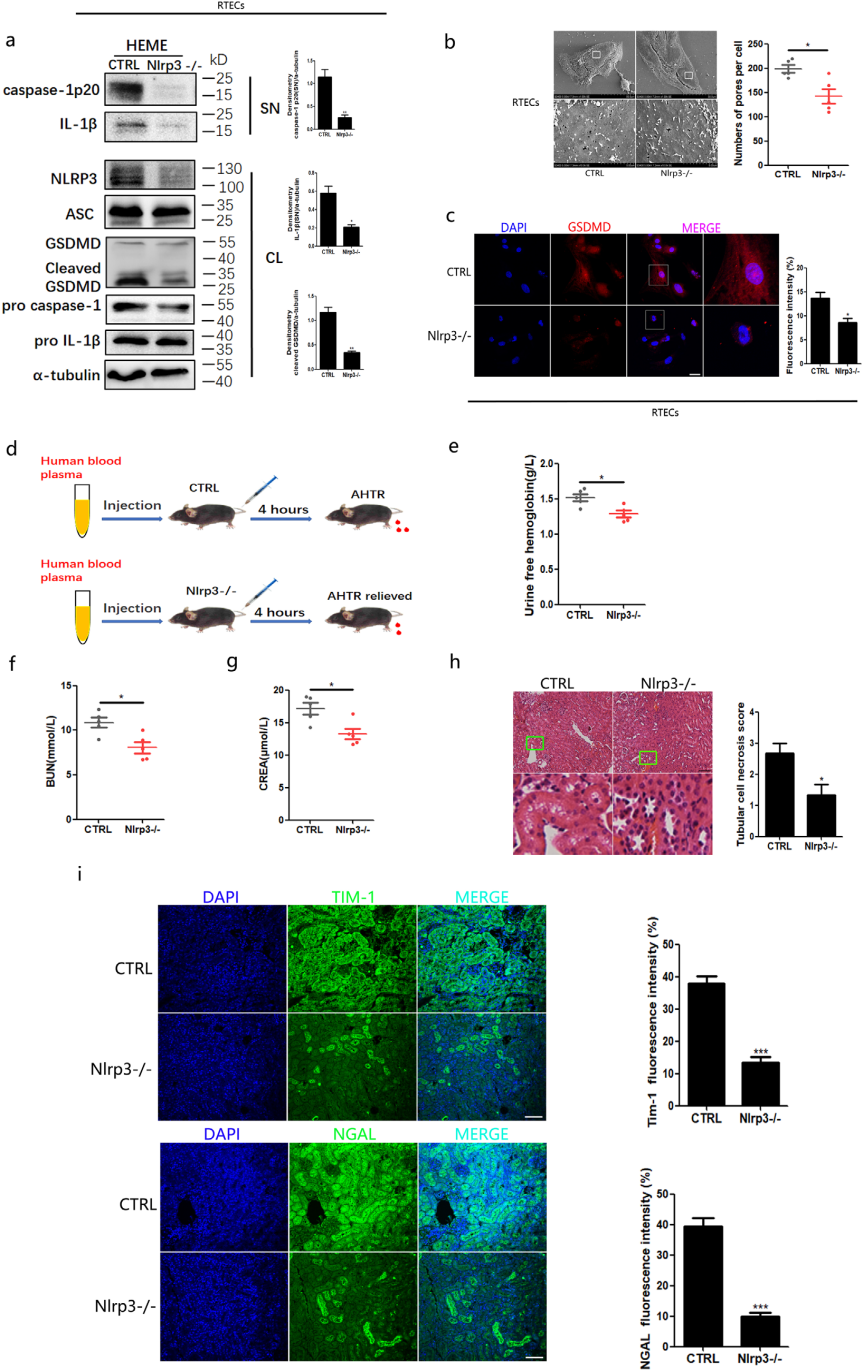
**Gene deficiency hindered the NLRP3 inflammasome activation of renal tubular epithelial cells of mice**. Primary RTECs from NLRP3^−/−^ and control mice were stimulated with chemical hemes for 4 h, following (A–C) analysis. A, Western blot analysis of indicated protein in total lysates (CL) and supernatants (SN) of NLRP3^−/−^RTECs and control mice primary RTECs. B, Electron microscopy scanning on NLRP3^−/−^RTECs and control mice primary RTECs (Magnification: the upper = 1000×; the lower = 1000×). The lower panels were enlarged from the white box of upper panel. C, The observation and analysis of nuclei (DAPI, blue) and GSDMD (green) foci of NLRP3^−/‐^ RTECs and control mice RTECs by laser confocal microscope (bar = 50μm). D, The animal model of hemolysis was established by the injection of human blood plasma. E, The urine was collected from each group and analyzed free hemoglobin. F–G, The analysis of blood urea nitrogen (BUN) and the creatinine (CREA). H, The H&E staining of kidney section from NLRP3^−/−^ and control hemolysis mice kidney section (bar = 200 μm), and the tubular cell necrosis scores. The lower panel was enlarged from the green box of upper panel to show the tubular damage region. I, The detection of TIM‐1 and NGAL by laser confocal microscope and quantity analysis of containing TIM‐1^+^ foci and NGAL^+^foci in NLRP3^−/−^ and control hemolysis mice kidney section (bar = 200μm). Each data represented data five mice per group. Data shown represent the mean ± SD from at least triplicate measurements. ^*^ *P* < .05; ^**^ *P* < .01; ^***^ *P* < .001

Considering that the NLRP3 inflammasome was activated in heme‐injured RTECs, we investigated whether NLRP3 gene deficiency could relieve kidney function injury due to hemes. Hence, we used NLRP3 knockout mice. We found that pro‐caspase‐1 was not cleaved in the primary RTECs from NLRP3 gene knockout mice after chemical hemes were administered (Figure 4A). Consistently, the levels of IL‐1β in NLRP3^−/−^ primary RTECs after chemical heme treatment were significantly lower than those in the controls (Figure 4A). The pyroptotic pores on the primary NLRP3^−/−^ RTEC surface were fewer than those on the control RTEC surface after chemical heme stimulation (Figure 4B). The GSDMD‐N immunofluorescence signals were also lower in NLRP3^−/‐^ RTECs after stimulation compared to those in the controls (Figure 4C). Based on these observations, we believe that the NLRP3 inflammasome could not be fully activated by heme in RTECs with NLRP3 gene deficiency.

Consequently, we investigated kidney function in AHTRs using NLRP3 gene‐deficient animal models (Figure 4D). We found that hemolysis was in remission in NLRP3^−/−^ mice with an AHTR based on the reduction in urine‐free hemoglobin levels (Figure 4E). BUN (Figure 4F) and CREA (Figure 4G) were also lower in the peripheral blood of hemolysis‐affected NLRP3^−/−^ mice than in that of control mice. When the kidneys were excised from mice and sectioned, H&E staining showed that more integrated tissue structures were maintained in the NLRP3 gene‐deficient mice, and the RTEC region necrosis scores were lower in the NLRP3^−/−^ group than in the control group (Figure 4H). Furthermore, immunofluorescence analysis indicated fewer TIM‐1 and NGAL signals in the tissue sections of NLRP3 gene‐deficient mice than those in the control group (Figure 4I). Taken together, these results demonstrated that NLRP3 gene knockout could partially prevent RTEC damage, which in turn recovered kidney function in the AHTR.

### 3.4 66PR could inhibit NLRP3 inflammasome activation in RTECs imposed by hemes

**FIGURE 5:**
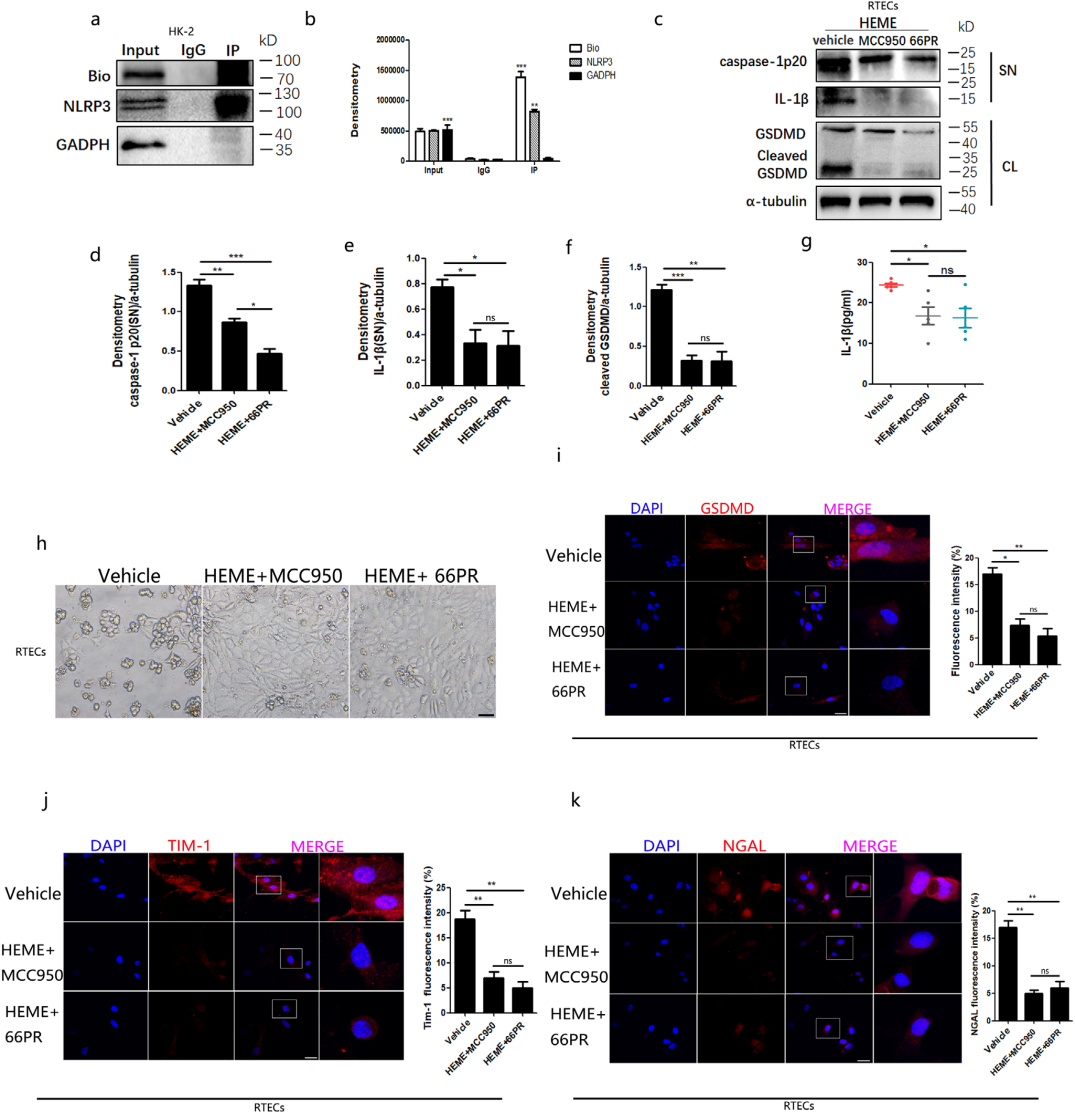
**The activation of NLRP3 inflammasome due to heme was inhibited by 66PR and MCC950 compound in renal tubular epithelial cells**. A,B, The 66PR compound was synthesized and linked with biotin. Then it was used to treat HK‐2 cells, and to detect the binding with NLRP3 by co‐Immunoprecipitation. C, Western blot analysis of NLRP3, Caspase‐1, GSDMD, and IL‐1β in total lysates (CL) and supernatants (SN) from primary RTECs after inhibitor treatment. D–F, Densitometry analysis ofcaspase‐1, IL‐1β, and GSDMD based on (C). G, ELISA analyses of IL‐1β release from primary RTECs supernatant after inhibitor administration. H, The observation of primary RTECs under light microscope after inhibitor treatment (bar = 50μm). I, The observation and analysis of nuclei (DAPI, blue) and GSDMD (red) foci of RTECs after inhibitor treatment by laser confocal microscope (bar = 50 μm). The rightmost panel was enlarged from the white box of neighbor panel. Fluorescence intensity (%) was the index of fluorescence, obtained by Image J software. J,K, The detection of TIM‐1 and NGAL by laser confocal microscope and quantity of primary RTECs containing TIM‐1^+^ foci and NGAL^+^foci after inhibitor treatment (bar = 50μm). The rightmost panel was enlarged from the white box of neighbor panel. Results are representative of five independent experiments. Data are shown as mean ± SD. ^*^ *P* < .05; ^**^ *P* < .01; ^***^ *P* < .001

Since NLRP3 gene deficiency benefited RTECs in hemolysis, we tried to refrain NLRP3 directly using chemical inhibitors. We previously reported that a compound named 66PR could inhibit NLRP3 inflammasome activation in mesenchymal stromal cells.^27^ We first computed the interaction between 66PR and NLRP3 protein by molecular docking. As shown in Figure S4, 66PR directly bound to the ATPase of NLRP3 NACHT domain to form a stable complex, which could inhibit the oligomerization and activation of NLRP3. We used co‐immunoprecipitation to test this prediction. We observed that 66PR could directly combined with NLRP3 (Figure 5A,B) in HK‐2 cells. These data indicated that 66PR could recognize and directly bind to the NLRP3 protein in renal tubular epithelial cells.

To investigate the effect of 66PR on NLRP3 inflammasome activation in RTECs, we stimulated primary RTECs with chemical hemes and 66PR together. MCC950 was used as the positive control for 66PR inhibition, which was a well‐defined chemical inhibitor of NLRP3 inflammasome.^25^ We found that caspase‐1p20 and IL‐1β in the supernatant of primary RTECs were decreased by 66PR and MCC950, according to western blot bands (Figure 5C–E). ELISA also showed that 66PR and MCC950 inhibited the secretion of IL‐1β in primary RTECs (Figure 5G). We further confirmed this finding in HK‐2 cells, and found that caspase‐1p20 and IL‐1β were also reduced by a chemical inhibitor (Figure 5A‐C). These results indicated that 66PR and MCC950 inhibited the activation of the NLRP3 inflammasome in RTECs and HK‐2 cells resulting from hemes in vitro. Moreover, we observed cellular injury remission after 66PR or MCC950 administration in primary RTECs (Figure 5H) and HK‐2 cells treated with chemical hemes (Figure S5E). At the same time, cell viability also increased after 66PR treatment (Figure S6D). The GSDMD‐N immunofluorescence signals were also lower in the chemical hemes and 66PR co‐cultured groups than in the chemical heme‐treated groups (Figure 5I; Figure S5F). Meanwhile, western blotting showed that cleaved GSDMD was obviously decreased by 66PR or MCC950 in both primary RTECs (Figure 5F) and HK‐2 cells (Figure S5D). These results indicated that 66PR inhibited heme‐induced pyroptosis, as well as MCC950. We then tested the effect of 66PR on the activation of the NLRP3 inflammasome and found that 66PR inhibited the outflow of K+ and ROS signals (Figure S6A,C), but it did not inhibit the mitochondria damage (Figure S6B). Altogether, these data revealed that 66PR could inhibit NLRP3 inflammasome activation resulting from hemes in renal tubular epithelial cells.

### 3.5 66PR recovered kidney function in hemolysis‐affected mice

Given that 66PR could inhibit NLRP3 inflammasome activation after hemes challenge, we investigated whether 66PR could relieve RTECs damage due to hemes. We found that fewer immunofluorescence signals of TIM‐1 (Figure 5J) and NGAL (Figure 5K) were observed in primary RTECs with chemical heme treatment after 66PR administration. When HK‐2 cells were employed, we observed a similar phenomenon (Figure S5G,H). These data demonstrate that 66PR could relieve RTEC damage from hemes.

**FIGURE 6:**
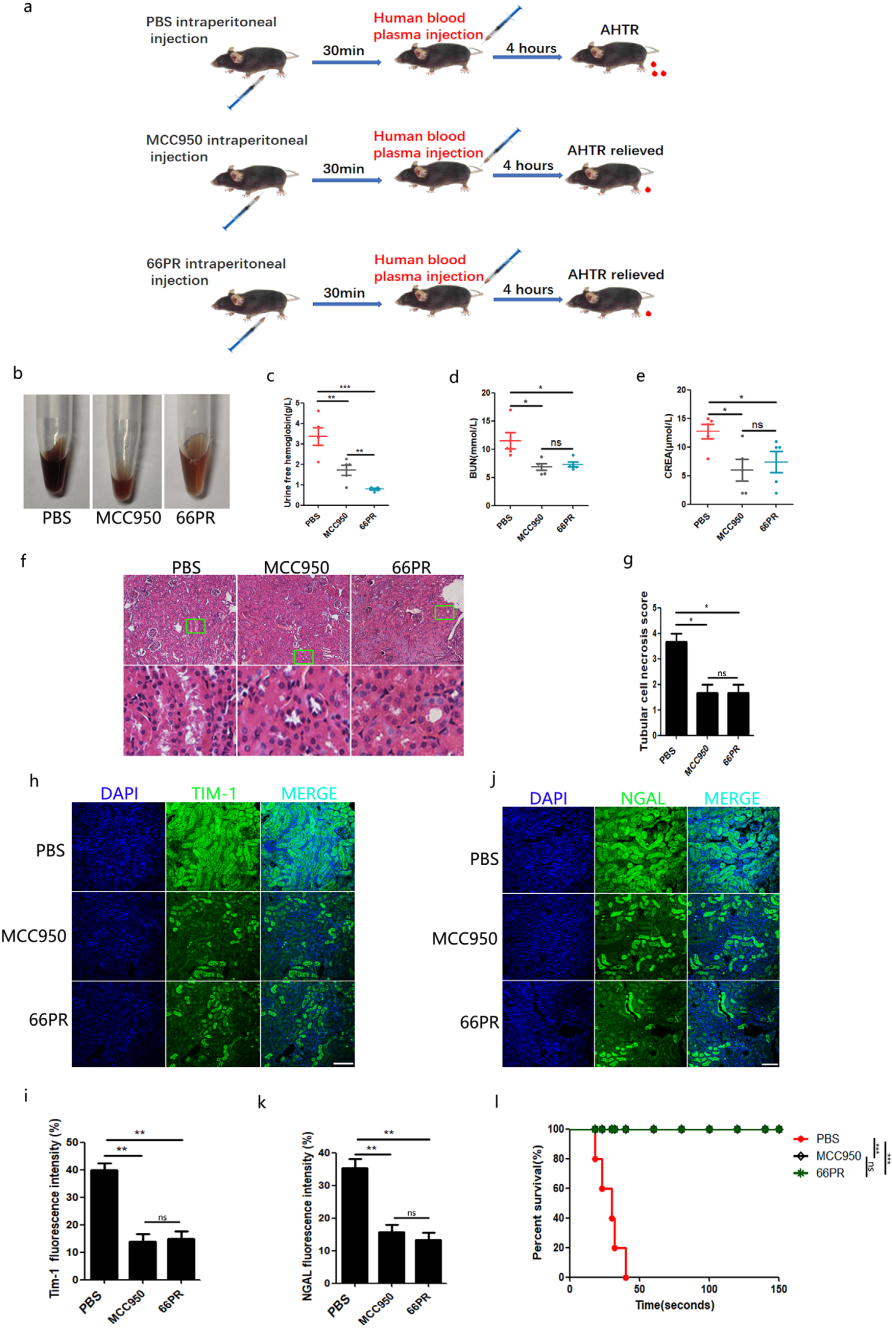
**66PR and MCC950 compound relieved the damage of kidney function in hemolysis mice**. A, The animal experimental scheme. B, The urine were collected from transfusion mice administrated with inhibitors. C, the analysis of free haemoglobin in urine of transfusion mice administrated with inhibitors. D‐E, The analysis of CREA and BUN in inhibitors administrated mice with transfusion. F, The H&E staining of kidney section from hemolysis mice injected with inhibitors (bar = 200μm). The lower panel was enlarged from the green box of upper panel to show the tubular damage region. G, The tubular cell necrosis scores, according to (F). H, The detection of TIM‐1 in kidney sections of hemolysis mice injected with inhibitors by laser confocal microscope (bar = 200μm). I, Quantity of kidney section containing TIM‐1+ foci. J, The detection of NGAL of kidney sections of hemolysis mice injected with inhibitors by laser confocal microscope (bar = 200μm). K, Quantity of laser confocal containing NGAL+ foci. L, The survival analysis of hemolysis mice with administration of inhibitors. Each experiment represents at least five mice per group. Data are shown as mean ± SD. ^*^ *P* < .05; ^**^ *P* < .01; ^***^ *P* < .001

We then wanted to determine whether 66PR could protect kidney function in the case of AHTRs. In the animal model of hemolysis (Figure 6A), we found that the free hemoglobin in the urine significantly decreased with 66PR treatment, suggesting that 66PR alleviated hemolysis (Figure 6B,C). We also tested BUN (Figure 6D) and CREA (Figure 6E) levels in the peripheral blood and found that the values were decreased in hemolysis‐affected mice after 66PR treatment . When the kidneys were excised from mice and sectioned, hematoxylin‐eosin staining revealed less injury to the tissues from the 66PR‐treated mice and the RTEC region necrosis scores were lower in the inhibitor group than in the control group (Figure 6F,G). Furthermore, immunofluorescence analysis showed weaker TIM‐1 (Figure 6H,I) and NGAL (Figure 6J,K) signals in the sections from 66PR‐treated hemolysis‐affected mice than in those of controls. Moreover, when were injected with human plasma at lethal doses, mice treated with 66PR or MCC950 survived, while the controls died in a short time (Figure 6L). Taken together, these data suggest that 66PR is helpful in the fight against kidney function impairment resulting from AHTRs, and the therapeutic efficacy of 66PR on AHTRs was much closer to that of MCC950.

## 4 DISCUSSION

In the current study, we found that hemes could damage RTECs by activating NLRP3 inflammasome both in vitro and in vivo. NLRP3 gene knockout could prevent damage to RTECs caused by hemes and recover kidney function in an AHTR. A novel chemical inhibitor named 66PR binds to the NLRP3 protein and inhibits inflammasome activation in RTECs, which consequently relieves kidney function injury caused by hemes. We believe that these findings are important for managing the toxic effects of heme on kidney function. Moreover, our findings were helpful in elucidating the mechanism of RTEC damage and the therapeutic target in AHTRs in more detail.

The classical concept of kidney function impairment resulting from AHTRs lies in renal tubule obstruction due to free hemoglobin crystals, microthrombosis of renal microvascular spasm, and tubular epithelial cell necrosis.^3^, ^7^, ^34^ Hemes produced in AHTRs come in contact with all cells in the kidney, including RTECs. Our finding that heme targets RTECs directly, which is consistent with previous studies reporting that hemes damage kidney tissue.^10^, ^11^ These findings are significant for the development of novel strategies targeting RTECs to prevent kidney function failure caused by hemes.

We revealed the role of NLRP3 inflammasomes in RTEC damage due to hemes. NLRP3 inflammasomes have been classically clarified the roles in immune cells such as macrophages, neutrophils, and dendritic cells.^35^, ^36^, ^37^ However, we, and other research groups, have previously confirmed that the NLRP3 inflammasome can be activated in non‐immune cells, including hepatic cells, skin cells, and mesenchymal stromal cells.^27^, ^29^, ^38^, ^39^, ^40^, ^41^, ^42^ We now provide evidence that NLRP3 inflammasome signaling also exists in RTECs, which further confirms that the NLRP3 inflammasome is an universal signal beyond immune cells. However, although hemes target various organs such as the liver, brain, and intestine, the detailed cellular and molecular mechanisms are not yet fully clarified, especially those in the kidney. Some researchers have reported that in kidney disease models, such as unilateral ureteral‐obstructed mice and chemical reperfusion‐injured mice, NLRP3 expression was augmented.^10^, ^11^, ^12^ However, more detailed information is not provided. Here, we proved that hemes activated the NLRP3 inflammasome and injured RTECs, resulting in kidney function failure. Therefore, our observations demonstrated the role of the NLRP3 inflammasome as a bridge between RTEC function failure and heme toxicity. IL‐1β is a major pro‐inflammatory factor of NLRP3 inflammasome activation. IL‐1α cleavage can also be induced by NLRP3 inflammasome stimuli,^43^ resulting in the co‐secretion of both IL‐1α and IL‐1β. It is unclear whether IL‐1α is involved in AHTRs. However, the receptors of IL‐1α and IL‐1β are same, IL‐1R. Therefore, IL‐1α and IL‐1β have similar downstream biological characteristics. Recombinant human IL‐1 receptor antagonists have been used to treat rheumatoid arthritis.^44^ Thus, recombinant human IL‐1 receptor antagonists may directly neutralize IL‐1β and ameliorate AHTRs. However, this requires further investigation in the future. Ours and other groups’ findings suggest that RTECs and the NLRP3 inflammasome are possible targets for developing new strategies for controlling kidney function failure.

Glibenclamide was reported to relieve chronic kidney diseases through NLRP3,^11^ which promoted the development of NLRP3 inflammasome inhibitors with small molecules. Based on cellular and molecular exploration, we used a previously developed NLRP3 inhibitor,^27^ 66PR. As expected, 66PR successfully countered RTEC damage due to hemes, which relieved kidney function damage in the AHTR murine models. This 66PR is not the only chemical NLRP3 inhibitor in development hitherto. Other NLRP3 inhibitors, including MCC950 and CY‐09, have been extensively studied.^25^, ^26^ MCC950, a type of thiourea compound, is a specific inhibitor of NLRP3 and has been once administered to hypertensive kidney and renal fibrosis induced by oxalate crystallization animal models.^45^, ^46^ The NLRP3 inhibition mechanism of MCC950 involves the binding of this compound to the Walker B motif of the NATCH domain and the prevention of hydrolysis, which decreases inflammasome packaging and activation. MCC950 can also change the conformation of NLRP3^24^, ^47^ and recognize the adenosine triphosphate motif of the NATCH domain to stop the packaging and activation of NLRP3 inflammasomes.^26^

Different from MCC950, 66PR is a compound of the 5‐FUMCL family derived from micheliolide (MCL). MCL, a sesquiterpene lactone isolated from *Magnoliaceae*, has anti‐inflammatory effects on intestinal inflammation, colitis‐associated cancer, rheumatoid arthritis, and diabetic nephropathy.^48^, ^49^ Moreover, N‐substituted pyrimidine nucleobases have attracted much interest because of their potential use as anti‐neoplastic, antiviral, and anticancer agents.^50^ The incorporation of fluorine into molecules is a powerful strategy in medicinal chemistry to modulate their hydrophobic character.^50^ Although the chemical structure of 66PR is completely different from that of MCC950, we found that both inhibitors could relieve RTEC damage and kidney function failure in AHTRs. The reason why these different kinds of inhibitors were effective in the hemolysis model therapy could not be determined in this study, but we believe that one possible explanation is their common target, NLRP3. According to the molecule docking, we predicated 66PR directly binds to the ATPase of NLRP3 NACHT domain, which is much similar to that of MCC950.^47^ But more details should be elucidated.

The current study has several limitations. One concern is that conditional NLRP3 gene knockout mice were not used here. The RTEC‐specific gene mutation animal model is not easily established because our group lacks a specific promoter. Moreover, a specific single marker of RTECs has not yet been fully defined. However, we used HK‐2 cells and primary murine RTECs in this study, which partially eliminated this limitation. Second, we only targeted the NLRP3 inflammasome, while other inflammasomes were not included in this study. We observed a small amount of cleaved IL‐1β even after NLRP3 gene knockout. One possible reason might be the presence of other inflammasomes in RTECs. We previously found that the caspase‐11 inflammasome was also inhibited by 66PR,^27^ which indicated that 66PR possibly inhibits inflammasomes other than NLRP3. Whether or not 66PR is a specific inhibitor of NLRP3 has not been reported in the present study. The specificity and binding site of NLRP3 should undoubtedly be verified in future, as the research in MCC950 or CY‐09. Although 66PR recovered kidney function failure induced by hemes through NLRP3 inhibition, we do not know whether the liver, lung, brain, and other organ function, as well as hematopoiesis, can be influenced or not. Once these concerns are clarified, the application of 66PR in the future will have a high potential. Inflammasome‐associated cell death includes both pyroptosis and apoptosis.^33^ For AHTRs, the renal tubular epithelial cells that suffer from apoptosis or pyroptosis require further investigation. Since delayed hemolytic transfusion reactions, hyperhemolysis, and passenger lymphocyte syndrome in transplant recipients are now becoming new challenges in current clinical transfusion practice, it is worth investigating whether NLPR3 also plays a role in these HTRs.

## 5 CONCLUSION

The present study clarified a novel mechanism by which hemes activate the NLRP3 inflammasome to damage RTECs, which leads to kidney function failure. The inhibition of NLRP3 inflammasome activation by a compound called 66PR relieved heme damage to the kidneys. We provided a new possible target to treat kidney function failure in AHTRs. Inhibition of the NLRP3 inflammasome may also be beneficial in other diseases, such as sickle anemia, β‐thalassemia, plasmodium infection, and rhabdomyolysis, which produce many hemes. Therefore, the NLRP3 inflammasome inhibition strategy has potential application prospects in protecting renal function.

## CONSENT FOR PUBLICATION

Each author approved the manuscript before submission for publication.

## AVAILABILITY OF DATA AND MATERIAL

The data used to support the findings of this study are available from the corresponding author upon request.

## AUTHOR CONTRIBUTIONS

Z.L. and Y.C. conducted most of the experiments. Y.C., Z.L., and X.H. wrote the manuscript. X.Q. synthesized the 66PR and bio‐66PRcompound. B.N. did the molecule docking. D.Y., F.F., S.G., J.X., N.A., J.Z., J.Y., and Q.A. helped to perform experiments. X.H. and X.Q. designed and supervised the study. W.Y. provided facilities and partially supervised the study.

## CONFLICT OF INTEREST

The authors have declared no conflict of interest.

## Supporting information

Figure S1 A and B, The agglutination in the tube from mice red blood cells reacted with human plasma and observed under light microscope (bar = 100μm). CTRTL: murine blood cells reacted with self‐plasma; HEMO: murine red blood cells reacted with human plasma. C, The detection of CK18, AQP1, VILLIN, and SGLT2 by laser confocal microscope (bar = 50μm); D, Quantity of percentage of positive renal tubular epithelial cells containing CK18, AQP1, VILLIN, and SGLT2. nuclei (DAPI, blue), CK18 (green), AQP1(green), VILLIN (green), SGLT2(green). At least 5 microscope fields were counted in each staining marker.

Figure S2 The model of hemolysis was established by human blood plasma transfusion, followed by (A‐J).Mice were transfused with human plasma (5μL/g) via caudal vein, as the group of hemolysis. The control group was injected with equal amount of PBS. After 4h, the peripheral blood and urine per group were collected and analyzed. HEMO: the group of hemolysis; CTRL: the control group. A, The analysis of red blood cells counts in peripheral blood. B, The analysis of free haemoglobin in peripheral blood. C, The observation of blood smears under light microscope (bar = 50μm). D, The analysis of poikilocytes count in blood smears. E, The direct Coomb's test after transfusion. MIgG: mono‐clonal anti‐human globulin; PcIgG: ploy clonal anti‐human globulin; C3d: complement 3d; NS: normal saline. F, The urine collected from each mouse in different group within 4 hours. G, The analysis of free haemoglobin in urine. (H‐J), The analysis of CREA, BUN and LDH in transfused mice peripheral blood. The model of hemolysis was established through injection of heme from murine destroyed RBCs, followed by (K‐M). Mice were injected with supernatant of lysated mice peripheral blood (10μL/g) via caudal vein, as HEMEs group. The control group was injected with equal amount of PBS. After 4h, the mice urines were collected and analyzed. HEME: the group of heme treatment; CTRL: the control group. K, The analysis of CREA in blood serum. L, The analysis of BUN in blood serum. M, The analysis of LDH in peripheral blood. N, Western blot analysis of TIM‐1 and NGAL protein in RTECs from hemolysis model mice with heme injection. Each data represents 5 mice per group are shown as mean±SD. *P < 0.05; **P < 0.01; ***P < 0.001.

Figure S3 A, The potassium (K+) concentration in the supernatant of HK‐2 cells after chemical hemes stimulation. B. HK‐2 cells were incubated with various concentrations of KCl for 15 minutes before stimulation with chemical heme to analyze IL‐1β maturation and caspase‐1p20 in cellular supernatants by western blot. C‐D, Densitometry analyses of Caspase1 and IL‐1β according to (B). E, HK‐2 cells were incubated with various concentrations of chemical heme for 4h to analyze mitochondrial membrane potential by flow cytometry. F, HK‐2 cells were incubated with various concentrations of chemical heme for 4h to analyze cellular ROS level by flow cytometry. G, HK‐2 cells were incubated with 50μM chemical heme and 25μM NAC for 4h to analyze cellular ROS level by flow cytometry. H, Cell viabilities were measured by CCK8 kit, after treated as in (G). Results were representative of five independent experiments. Data are shown as mean±SD. *P < 0.05; ** P < 0.01; *** P < 0.001.

Figure S4 ATPase lies in NLRP3 NACHT domain. A, The 2‐dimensional projection plan of the non‐bonding interaction between 66PR and ATPase. B, The diagram of hydrogen bonding interaction between 66PR and amino acid residues (GLY229, LYS230, THR231, Ile232, Arg235) in ATPase; C, The diagram shows the hydrophobic interaction between 66PR and amino acid residues (LEU411, PRO410, TRP414, HIS520) in ATPase.

Figure S5 A, Western blot analysis of caspase‐1, GSDMD and IL‐1β in total lysates (CL) and supernatants (SN) from HK‐2 cells after inhibitors treatment. B‐D, Densitometry analysis of IL‐1β, Caspase‐1 and GSDMD based on (A). E, The observation of HK‐2 cells under light microscope after inhibitors administration (bar = 50μm). F, The observation and analysis of nuclei (DAPI, blue) and GSDMD (red) foci of HK‐2cells after inhibitors treatment by laser confocal microscope (bar = 50μm). G‐H, The detection of TIM‐1 and NGAL by laser confocal microscope and quantity of HK‐2cells containing TIM‐1^+^ foci and NGAL ^+^ foci after inhibitors treatment (bar = 50μm). Results are representative of five experiments. Data are shown as mean±SD. *P < 0.05; ** P < 0.01; *** P < 0.001.

Figure S6 HK‐2 cells were incubated 66PR compound and 50μM chemical hemes for 4h. A, The potassium (K+) concentration in the supernatant of HK‐2 cells were measured. B, The mitochondrial membrane potential was measured by flow cytometry. C, Cellular ROS levels were detected by flow cytometry. D, Cell viabilities were measured by CCK8 kit. Results are representative of five independent experiments. Data are shown as mean±SD.*P < 0.05; ** P < 0.01; *** P < 0.001, ns: no significant.
