# Supplementary figures and images for: NLRP3 inflammasome of renal tubular epithelial cells induces kidney injury in acute hemolytic transfusion reactions
Source: Clin Transl Med. 2021 Mar 30;11(3):e373. doi: 10.1002/ctm2.373 (PMC8009139; doi:10.1002/ctm2.373)

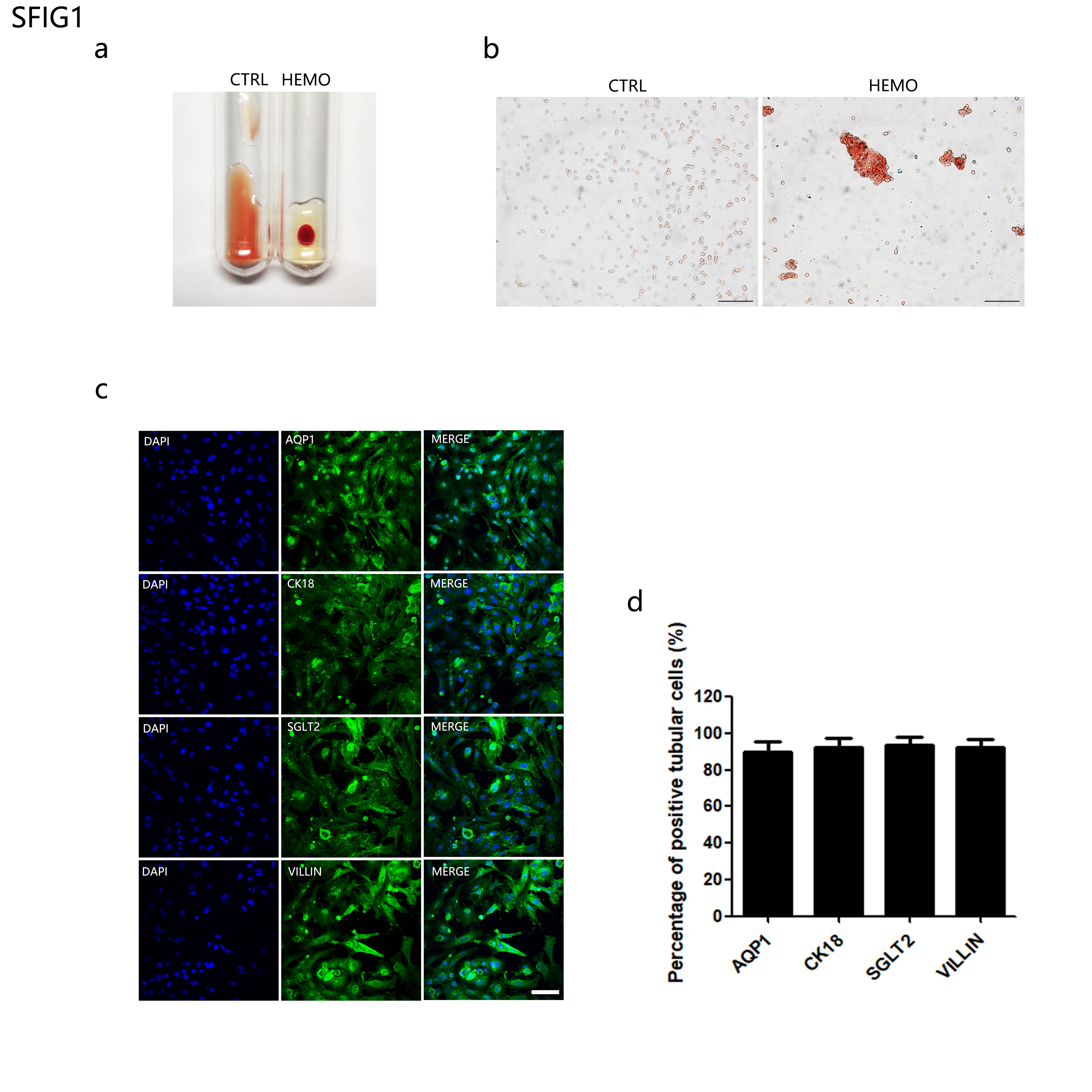

Supplement: Supplementary file 1 — Figure S1 A and B, The agglutination in the tube from mice red blood cells reacted with human plasma and observed under light microscope (bar = 100μm). CTRTL: murine blood cells reacted with self‐plasma; HEMO: murine red blood cells reacted with human plasma. C, The detection of CK18, AQP1, VILLIN, and SGLT2 by laser confocal microscope (bar = 50μm); D, Quantity of percentage of positive renal tubular epithelial cells containing CK18, AQP1, VILLIN, and SGLT2. nuclei (DAPI, blue), CK18 (green), AQP1(green), VILLIN (green), SGLT2(green). At least 5 microscope fields were counted in each staining marker. [file CTM2-11-e373-s003.jpg]

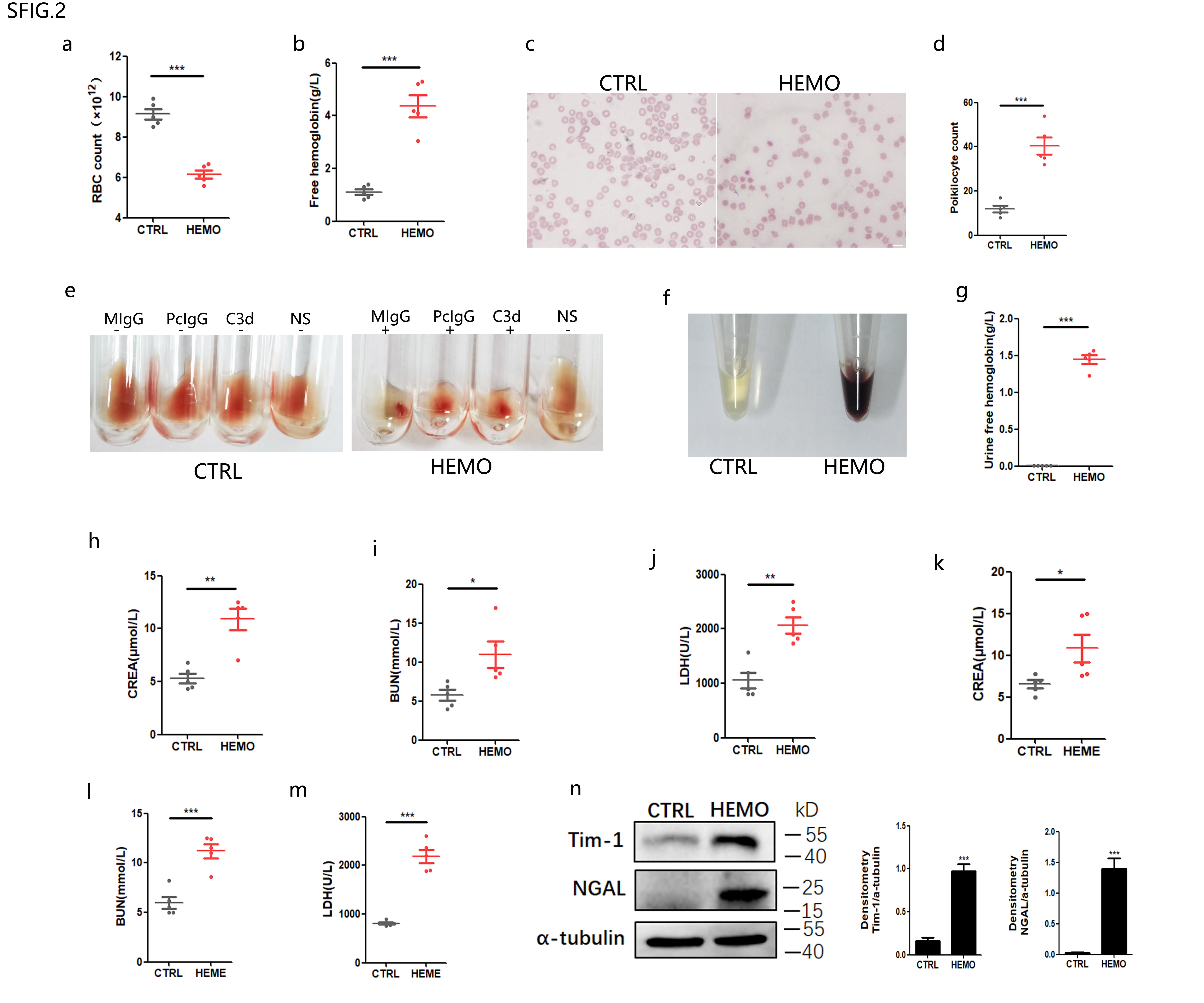

Supplement: Supplementary file 2 — Figure S2 The model of hemolysis was established by human blood plasma transfusion, followed by (A‐J).Mice were transfused with human plasma (5μL/g) via caudal vein, as the group of hemolysis. The control group was injected with equal amount of PBS. After 4h, the peripheral blood and urine per group were collected and analyzed. HEMO: the group of hemolysis; CTRL: the control group. A, The analysis of red blood cells counts in peripheral blood. B, The analysis of free haemoglobin in peripheral blood. C, The observation of blood smears under light microscope (bar = 50μm). D, The analysis of poikilocytes count in blood smears. E, The direct Coomb's test after transfusion. MIgG: mono‐clonal anti‐human globulin; PcIgG: ploy clonal anti‐human globulin; C3d: complement 3d; NS: normal saline. F, The urine collected from each mouse in different group within 4 hours. G, The analysis of free haemoglobin in urine. (H‐J), The analysis of CREA, BUN and LDH in transfused mice peripheral blood. The model of hemolysis was established through injection of heme from murine destroyed RBCs, followed by (K‐M). Mice were injected with supernatant of lysated mice peripheral blood (10μL/g) via caudal vein, as HEMEs group. The control group was injected with equal amount of PBS. After 4h, the mice urines were collected and analyzed. HEME: the group of heme treatment; CTRL: the control group. K, The analysis of CREA in blood serum. L, The analysis of BUN in blood serum. M, The analysis of LDH in peripheral blood. N, Western blot analysis of TIM‐1 and NGAL protein in RTECs from hemolysis model mice with heme injection. Each data represents 5 mice per group are shown as mean±SD. *P < 0.05; **P < 0.01; ***P < 0.001. [file CTM2-11-e373-s004.jpg]

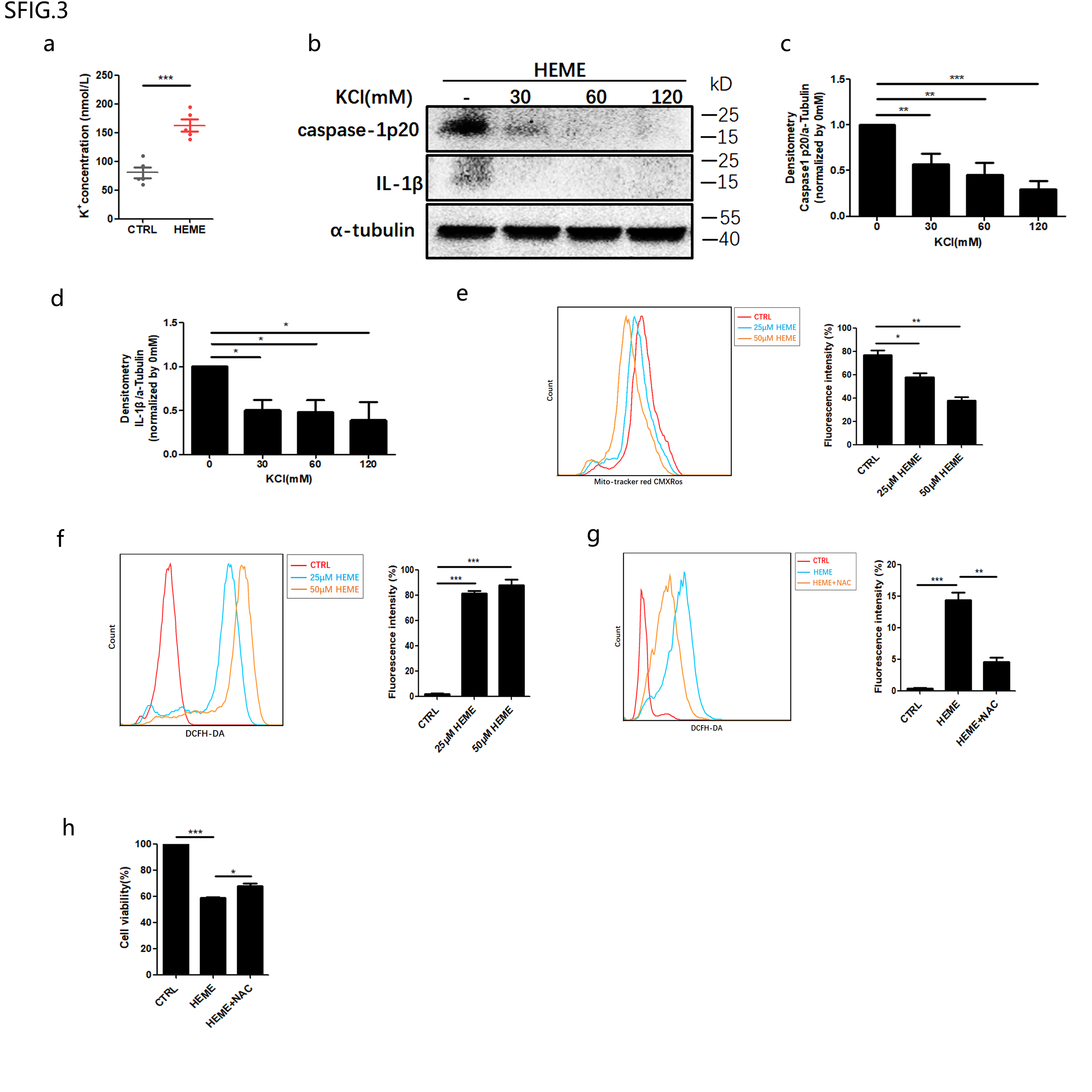

Supplement: Supplementary file 3 — Figure S3 A, The potassium (K+) concentration in the supernatant of HK‐2 cells after chemical hemes stimulation. B. HK‐2 cells were incubated with various concentrations of KCl for 15 minutes before stimulation with chemical heme to analyze IL‐1β maturation and caspase‐1p20 in cellular supernatants by western blot. C‐D, Densitometry analyses of Caspase1 and IL‐1β according to (B). E, HK‐2 cells were incubated with various concentrations of chemical heme for 4h to analyze mitochondrial membrane potential by flow cytometry. F, HK‐2 cells were incubated with various concentrations of chemical heme for 4h to analyze cellular ROS level by flow cytometry. G, HK‐2 cells were incubated with 50μM chemical heme and 25μM NAC for 4h to analyze cellular ROS level by flow cytometry. H, Cell viabilities were measured by CCK8 kit, after treated as in (G). Results were representative of five independent experiments. Data are shown as mean±SD. *P < 0.05; ** P < 0.01; *** P < 0.001. [file CTM2-11-e373-s001.jpg]

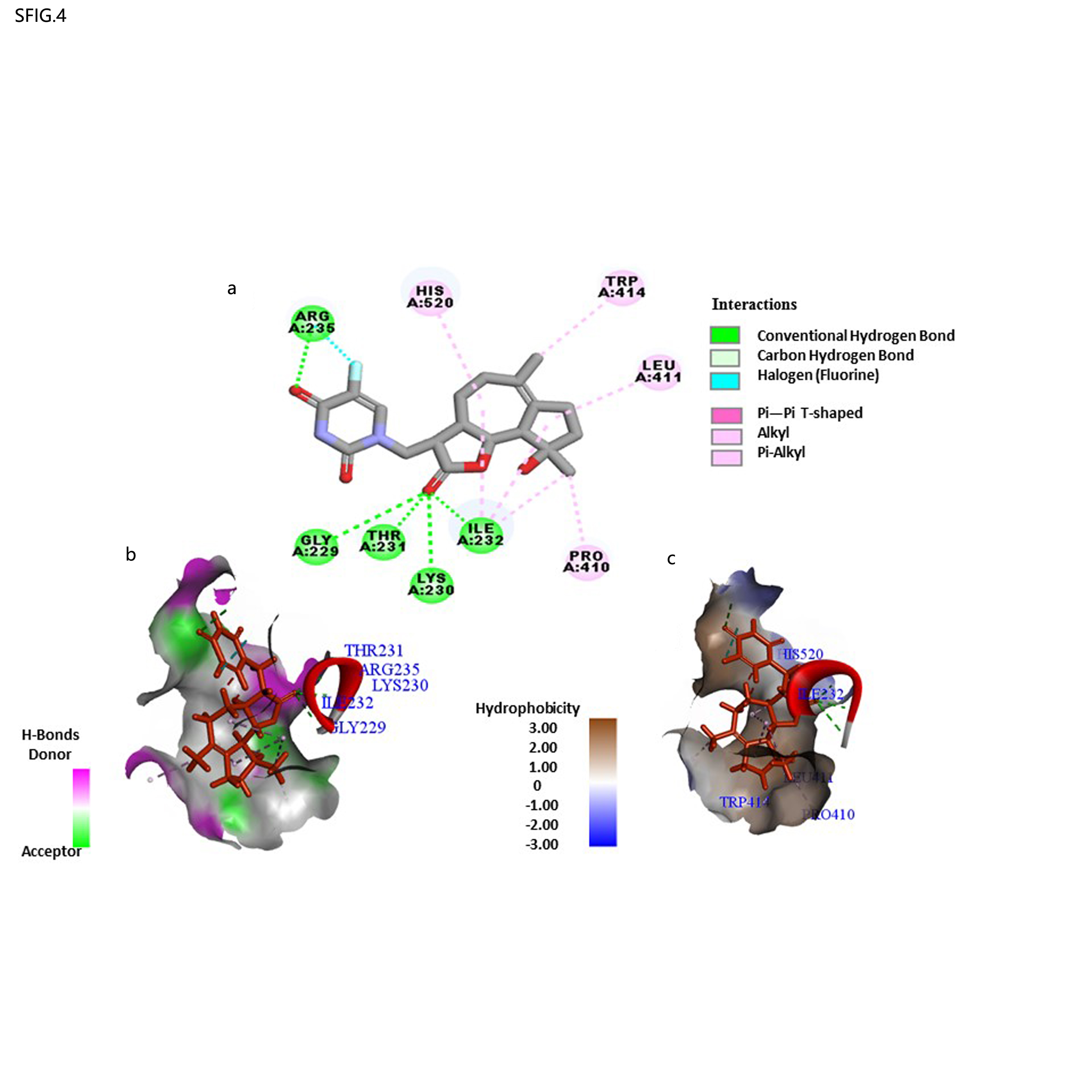

Supplement: Supplementary file 4 — Figure S4 ATPase lies in NLRP3 NACHT domain. A, The 2‐dimensional projection plan of the non‐bonding interaction between 66PR and ATPase. B, The diagram of hydrogen bonding interaction between 66PR and amino acid residues (GLY229, LYS230, THR231, Ile232, Arg235) in ATPase; C, The diagram shows the hydrophobic interaction between 66PR and amino acid residues (LEU411, PRO410, TRP414, HIS520) in ATPase. [file CTM2-11-e373-s006.jpg]

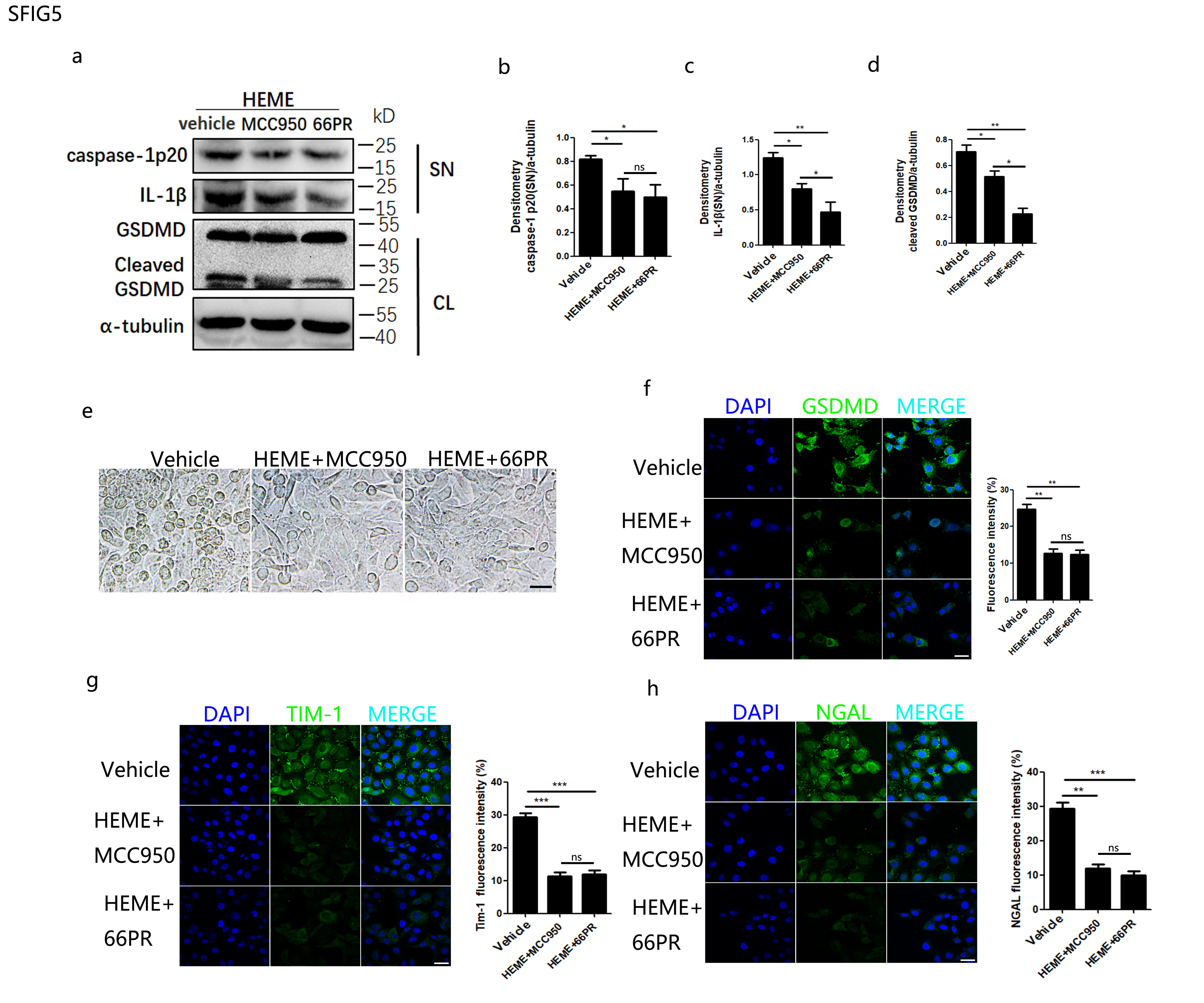

Supplement: Supplementary file 5 — Figure S5 A, Western blot analysis of caspase‐1, GSDMD and IL‐1β in total lysates (CL) and supernatants (SN) from HK‐2 cells after inhibitors treatment. B‐D, Densitometry analysis of IL‐1β, Caspase‐1 and GSDMD based on (A). E, The observation of HK‐2 cells under light microscope after inhibitors administration (bar = 50μm). F, The observation and analysis of nuclei (DAPI, blue) and GSDMD (red) foci of HK‐2cells after inhibitors treatment by laser confocal microscope (bar = 50μm). G‐H, The detection of TIM‐1 and NGAL by laser confocal microscope and quantity of HK‐2cells containing TIM‐1+ foci and NGAL + foci after inhibitors treatment (bar = 50μm). Results are representative of five experiments. Data are shown as mean±SD. *P < 0.05; ** P < 0.01; *** P < 0.001. [file CTM2-11-e373-s002.jpg]

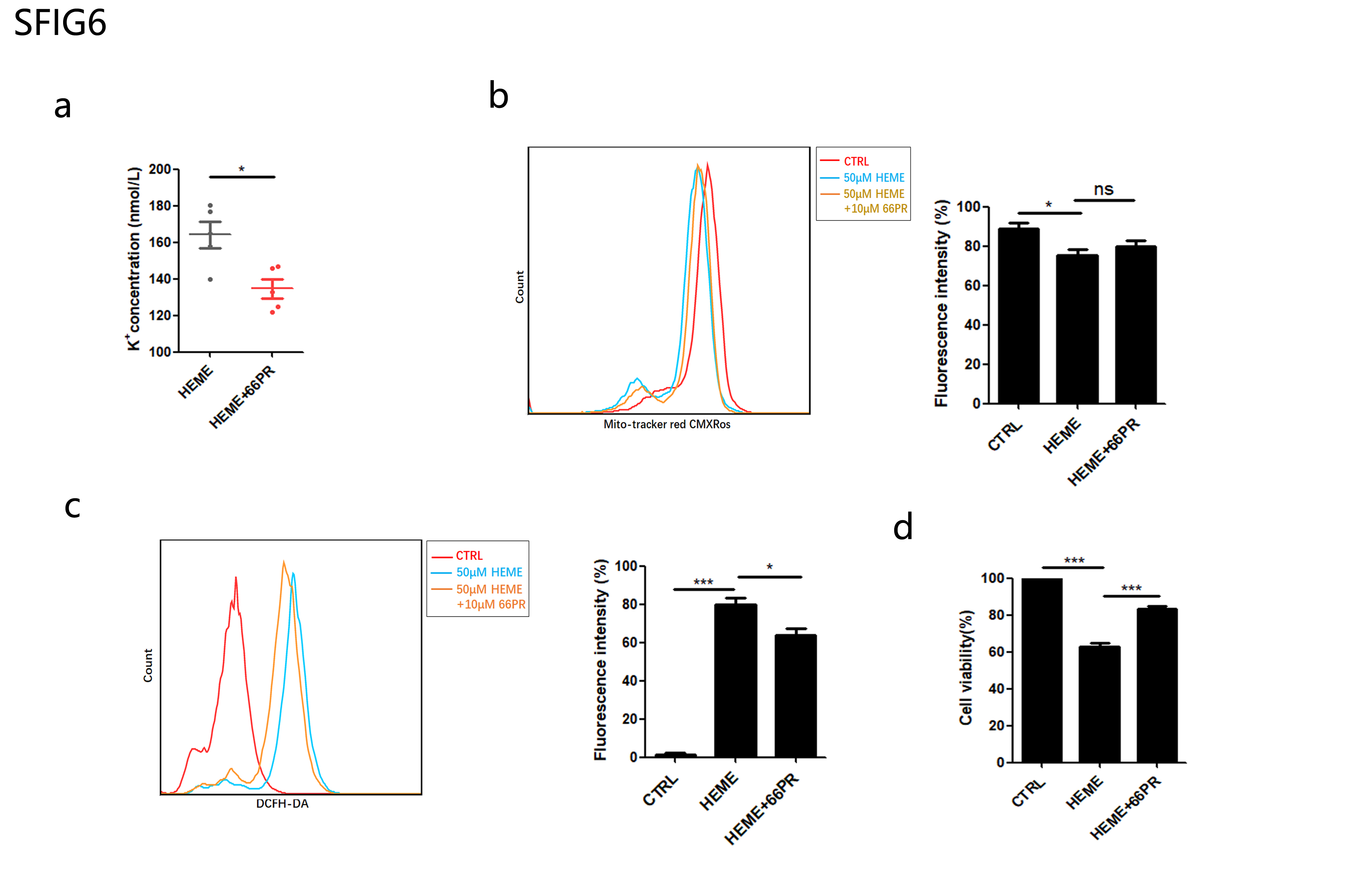

Supplement: Supplementary file 6 — Figure S6 HK‐2 cells were incubated 66PR compound and 50μM chemical hemes for 4h. A, The potassium (K+) concentration in the supernatant of HK‐2 cells were measured. B, The mitochondrial membrane potential was measured by flow cytometry. C, Cellular ROS levels were detected by flow cytometry. D, Cell viabilities were measured by CCK8 kit. Results are representative of five independent experiments. Data are shown as mean±SD.*P < 0.05; ** P < 0.01; *** P < 0.001, ns: no significant. [file CTM2-11-e373-s005.jpg]
